# Pharmacokinetics of Caffeine: A Systematic Analysis of Reported Data for Application in Metabolic Phenotyping and Liver Function Testing

**DOI:** 10.3389/fphar.2021.752826

**Published:** 2022-02-25

**Authors:** Jan Grzegorzewski, Florian Bartsch, Adrian Köller, Matthias König

**Affiliations:** Institute for Theoretical Biology, Humboldt-University Berlin, Berlin, Germany

**Keywords:** caffeine, pharmacokinetics, smoking, oral contraceptives, drug-drug interactions, drug-disease interactions, CYP1A2, liver function test

## Abstract

Caffeine is by far the most ubiquitous psychostimulant worldwide found in tea, coffee, cocoa, energy drinks, and many other beverages and food. Caffeine is almost exclusively metabolized in the liver by the cytochrome P-450 enzyme system to the main product paraxanthine and the additional products theobromine and theophylline. Besides its stimulating properties, two important applications of caffeine are metabolic phenotyping of cytochrome P450 1A2 (CYP1A2) and liver function testing. An open challenge in this context is to identify underlying causes of the large inter-individual variability in caffeine pharmacokinetics. Data is urgently needed to understand and quantify confounding factors such as lifestyle (e.g., smoking), the effects of drug-caffeine interactions (e.g., medication metabolized via CYP1A2), and the effect of disease. Here we report the first integrative and systematic analysis of data on caffeine pharmacokinetics from 141 publications and provide a comprehensive high-quality data set on the pharmacokinetics of caffeine, caffeine metabolites, and their metabolic ratios in human adults. The data set is enriched by meta-data on the characteristics of studied patient cohorts and subjects (e.g., age, body weight, smoking status, health status), the applied interventions (e.g., dosing, substance, route of application), measured pharmacokinetic time-courses, and pharmacokinetic parameters (e.g., clearance, half-life, area under the curve). We demonstrate via multiple applications how the data set can be used to solidify existing knowledge and gain new insights relevant for metabolic phenotyping and liver function testing based on caffeine. Specifically, we analyzed 1) the alteration of caffeine pharmacokinetics with smoking and use of oral contraceptives; 2) drug-drug interactions with caffeine as possible confounding factors of caffeine pharmacokinetics or source of adverse effects; 3) alteration of caffeine pharmacokinetics in disease; and 4) the applicability of caffeine as a salivary test substance by comparison of plasma and saliva data. In conclusion, our data set and analyses provide important resources which could enable more accurate caffeine-based metabolic phenotyping and liver function testing.

## 1 Introduction

Caffeine is commonly found in tea, coffee, cocoa, energy drinks, and many other beverages. It is by far the most ubiquitous psychostimulant worldwide (Gilbert, 1984), with 85% of the United States population consuming caffeine daily (Mitchell et al., 2014). Among caffeine consumers, the average consumption is more than 200 mg of caffeine per day (Frary et al., 2005). Caffeine is mainly known for its stimulating properties but is also consumed for improved exercise performance and the treatment of various diseases (e.g., apnea in prematurity, hypersomnia). Two important applications of caffeine are liver function testing and metabolic phenotyping of cytochrome P450 1A2 (CYP1A2), N-acetyltransferase 2 (NA2), and xanthine oxidase (XO) (Wang et al., 1985; Jost et al., 1987; Wahlländer et al., 1990; Tripathi et al., 2015).

Caffeine is almost exclusively metabolized in the liver by the cytochrome P450 enzyme system with 3% or less being excreted unchanged in urine (Kot and Daniel, 2008). In humans, N-3 demethylation of caffeine (1,3,7-trimethylxanthine) to paraxanthine (1,7-dimethylxanthine) is the main reaction in the metabolism of caffeine, accounting for around 80–90% of caffeine demethylation. The reaction is exclusively mediated by the activity of the cytochrome P450 isoform 1A2 (CYP1A2) (Hakooz, 2009). The remainder of caffeine is metabolized to around 11 and 4% to the 1-demethylated product theobromine and 7-demethylated product theophylline, respectively (Lelo et al., 1986b; Kalow and Tang, 1993; Miners and Birkett, 1996; Amchin et al., 1999).

Large variation exists in the consumption of caffeine-containing beverages and food between individuals, which can induce CYP1A2 activity. In addition, CYP1A2 activity and protein amount are affected by environmental, genetic, and epigenetic factors (Klein et al., 2010) resulting in large variation between 5-6 fold in humans (Schrenk et al., 1998). These factors lead to a wide range of caffeine plasma concentrations and caffeine pharmacokinetics.

Sex does not significantly influence the CYP1A2 activity (Klein et al., 2010; Yang et al., 2010; Puri et al., 2020). A large heritability of CYP1A2 activity could be shown by two twin studies (Rasmussen et al., 2002; Matthaei et al., 2016). With excluded users of hormonal contraceptives and smokers, 89% of the variation in caffeine AUC was shown to be due to genetic effects and 8% specifically due to the CYP1A1/1A2 promoter polymorphism. In other studies, statistically significant genetic or epigenetic markers on the CYP1A locus on chromosome 15 could not be found (Moonen et al., 2004; Jiang et al., 2006; Ghotbi et al., 2007; Gunes and Dahl, 2008; Myrand et al., 2008; Klein et al., 2010; Yang et al., 2010). Genes regulating the expression and function of CYP1A2 and non-genetic factors could explain 42, 38, and 33% of CYP1A2 variation at activity, protein, and mRNA level, respectively (Klein et al., 2010). Lifestyle factors (e.g., smoking) and use of oral contraceptives have been shown to influence caffeine pharmacokinetics, as have pregnancy, obesity, alcohol consumption, and the coadministrations of drugs (e.g., fluvoxamine and pipmedic acid). Many diseases reduce the metabolic capabilities of patients. For caffeine which is predominantly metabolized by the liver, various liver diseases result in a strong reduction in caffeine clearance. The most profound reduction is observed in cirrhotic liver disease, correlating with the degree of hepatic impairment (Holstege et al., 1989; Park et al., 2003; Jodynis-Liebert et al., 2004; Tripathi et al., 2015).

Metabolic phenotyping of enzymes by probe drugs is a common method to evaluate the impact of lifestyle, drug-gene and drug-drug interactions, and other factors influencing enzyme activity. Caffeine is an established probe drug for CYP1A2, N-acetyltransferase 2 (NAT2), and xanthine oxidase (XO) metabolic activities (Fuhr et al., 1996; Miners and Birkett, 1996; Faber et al., 2005; Hakooz, 2009). It is rapidly and completely absorbed by the gastrointestinal tract, distributed throughout the total body water, has low plasma binding, as well as short half-life, negligible first-pass metabolism (Yesair et al., 1984), minimal renal elimination, excellent tolerability, and its biotransformation is virtually confined to the liver (Kalow and Tang, 1993; Amchin et al., 1999; Drozdzik et al., 2018). Caffeine is especially used for CYP1A2 phenotyping which contributes 5–20% to the hepatic P450 pool and is involved in the clearance of about 9% of clinically used drugs (Zanger and Schwab, 2013). The partial or systemic caffeine clearance measured in plasma is considered to be the gold standard for CYP1A2 phenotyping (Fuhr et al., 1996) since 95% of the systemic clearance of caffeine is estimated to be due to hepatic CYP1A2 (Amchin et al., 1999). Measurements in serum, saliva, and urine are extensively studied as well. In urine, sampling at multiple time points and precise timing are inherently difficult. Thus, clearance rates are typically calculated only from plasma, saliva, and serum samples. Measurements in saliva are not invasive and show good correlation with measurements in plasma (Callahan et al., 1982; Wahlländer et al., 1990). The metabolic ratio (MR) between various metabolites of caffeine is an established alternative measure for CYP1A2 enzyme activity (Hakooz, 2009). Analogously, the MR is measured in any of the above mentioned tissues though typically only at a single time point after drug administration. The MR of various metabolites at 4 h after dosing in plasma, saliva, and urine correlate well with the apparent caffeine clearance, 0.84, 0.82, 0.61, respectively (Carrillo et al., 2000). The MRs measured in plasma and urine have been historically most popular. However, measurements in saliva are routinely applied, especially in epidemiological studies (Tantcheva-Poór et al., 1999; Kukongviriyapan et al., 2004; Tripathi et al., 2015; Chia et al., 2016; Urry et al., 2016; Puri et al., 2020).

Despite the great potential of caffeine as a test substance for liver function tests and CYP1A2 based phenotyping, so far caffeine testing has not found widespread clinical adoption. For liver function tests, a major limiting factor is the large inter-individual variability. Data is urgently needed to understand and quantify confounding factors of caffeine pharmacokinetics such as lifestyle (e.g., smoking) and the effects of drug-drug interactions (e.g., drugs metabolized via CYP1A2) or how disease alters caffeine elimination. Based on such data, more accurate liver function tests and CYP1A2 phenotyping protocols could be established. Differences in clinical protocols (e.g., dosing amount, sampling tissue, and timing) have not been systematically analyzed in the literature. In addition, data on competing substances in the context of dynamical liver function tests (e.g., metacethin used in the LiMAx test (Rubin et al., 2017)) and CYP1A2 phenotyping is not accessible but absolutely imperative for a quantitative evaluation of these methods.

Caffeine pharmacokinetics have been investigated in a multitude of clinical trials, each with its own focus and research question. These studies have been reviewed in a broad scope, most recently in (Arnaud, 2011; Nehlig, 2018). Despite caffeine pharmacokinetics being highly studied in literature, no integrated pharmacokinetics data set exists so far and no systematic analysis of the reported data has been performed. The objective of this work was to fill this gap by providing the first comprehensive high-quality data set of reported data on caffeine pharmacokinetics and demonstrate its value via multiple example applications relevant for metabolic phenotyping and liver function testing based on caffeine.

## 2 Materials and Methods

**FIGURE 1:**
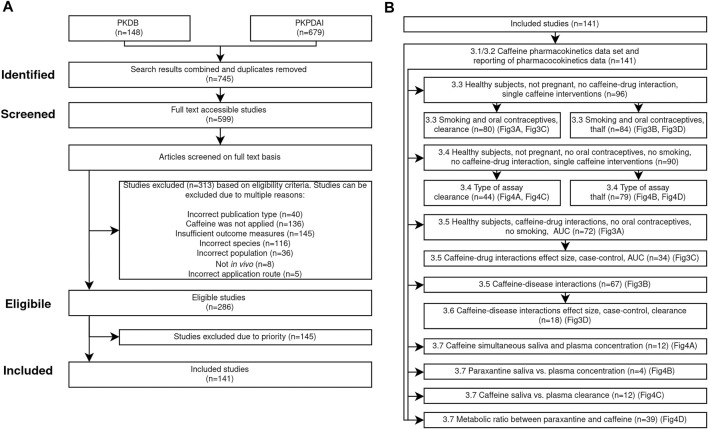
PRISMA flow diagram. **(A)** Overview of search strategy and inclusion/exclusion criteria applied in the systematic analysis of pharmacokinetics of caffeine. The applied workflow resulted in *n* = 141 included studies. **(B)** Subsets of the included studies were used for the various analyses. For details see the Section 2.

This study is guided by the Preferred Reporting Items for Systematic reviews and Meta-Analysis (PRISMA) statement and its extension for Scoping Reviews (PRISMA-ScR) (Liberati et al., 2009; Tricco et al., 2018). This work was conducted with the aim to answer the following research questions. 1) What is the current state of research on caffeine pharmacokinetics in adult humans in the context of metabolic phenotyping and liver function testing? 2) How homogeneous is the reporting? 3) How do caffeine dose and route impact caffeine pharmacokinetics? 4) What is the effect of smoking and oral contraceptive use on caffeine pharmacokinetics with respect to the caffeine dose? 5) What is the effect of coadministrations on caffeine pharmacokinetics with respect to the dosing amount and what are the effect sizes of caffeine-drug interactions? 6) What is the effect of diseases on caffeine pharmacokinetics with respect to the caffeine dose and what are the effect sizes of caffeine-disease interactions? 7) How do sampling time and tissue influence pharmacokinetics and phenotyping? The search, screening and filtering process are depicted in the PRISMA flow diagram in Figure 1.

### 2.1 Search Strategy

We searched the general purpose pharmacokinetics database PK-DB with the search query https://pk-db.com/api/v1/filter/?concise=false&download=true&interventions__substance=caffeine on 2021–10–06 and PKPDAI (Hernandez et al., 2021) with the search query https://app.pkpdai.com/?term=caffeine on 2021–10–06. PKPDAI is a dedicated search engine for pharmacokinetics data based on PubMed. This search resulted in 745 studies from which 599 could be accessed as full text.

### 2.2 Eligibility Criteria

Studies were included or excluded based on the following eligibility criteria. Reviews or publications describing computational models were excluded. For a study to be eligible caffeine must be applied, it must report pharmacokinetics data for caffeine or its metabolites, data must be measured in humans, and individuals and groups must consisted of adults (age >= 18 years), and data must be *in vivo* data. The application route of caffeine must be either oral, intravenous, or intramuscular. All application forms of caffeine (e.g., tablet, capsule, solution) are eligible. No restrictions were imposed on the dosing amount of caffeine or coadministrations. Relevant outcome measures are concentration-time profiles in plasma, serum, blood, and saliva of caffeine and metabolites and corresponding pharmacokinetic parameters. Studies containing pharmacokinetic parameters of caffeine and metabolites (i.e., clearance, maximum concentration, time of maximum concentration, half-life, AUC) and metabolic ratios of caffeine and its metabolites were included. In total 286 studies met our eligibility criteria, 313 studies were excluded for various reasons as described in Figure 1. Of the 286 studies, 145 were excluded due to low priority, e.g., if only urinary data was reported. Data was extracted and curated from the remaining 141 studies. During the curation process outliers from four studies (Stille et al., 1987; Harder et al., 1988, 1989; Balogh et al., 1992) were identified and excluded from all subsequent analyses. All four studies probably originate from the same clinical investigation.

### 2.3 Data Curation

Pharmacokinetics data was curated manually as part of the pharmacokinetics database PK-DB (https://pk-db.com/) (Grzegorzewski et al., 2020) using established workflows. The pharmacokinetics data was stored in combination with relevant metadata on groups, individuals, interventions, and outputs. PK-DB provided support in the curation process with strong validation rules, unit normalization, and automatic calculation of pharmacokinetic parameters from time-courses. As part of the curation process and the presented analyses, pharmacokinetic parameters and other commonly reported measurements were integrated from multiple studies. The meta-analyses and data integration allowed to identify and correct/remove outlier data which were mostly due to either curation errors or incorrect reporting. Pharmacokinetic parameters calculated from time-courses are included in the analyses. For more details see (Grzegorzewski et al., 2020).

### 2.4 Data Processing and Filtering

In Figures 3A,B, Figures 5A–D, and Figure 4 the data is displayed in a similar manner. For collectively reported subjects, the group size and standard error is displayed as the marker size and error-bar, respectively. In the legend, (I), (G), and (TI) stand for individual participant data, the number of groups, and the total number of subjects, respectively. Data points are depicted as circles if reported equivalently in the source, as squares if calculated from concentration-time profiles and as triangles if inferred from corresponding pharmacokinetic data and body weights of the subjects. Typically, dosing is reported in mass units, AUC in mass per volume units, clearance in volume per time units, and half-life in time units. Occasionally, dosage, AUC, and clearance are reported in body weight units. In case of reported subject weight, the data is harmonized to similar units by multiplying with the reported weights.

In Figure 3, the depicted subjects were healthy. Male subjects were assumed to not take any oral contraceptives. Substances with negligible caffeine-drug interactions were determined by an effect size analysis in Section 3.5. All other co-administrations and investigations containing multiple caffeine dosages were excluded.

In subplot Figure 5A, included subjects were healthy. The area under the caffeine concentration curves measured at least up to 12 h after a single application of caffeine and AUC extrapolated to infinity were included. Multiple subsequent caffeine dosages were excluded, other administrations and co-administrations included. In subplot Figure 5C, healthy and non-healthy subjects were included. Single applications of caffeine or caffeine administrated as a cocktail with negligible drug-drug interactions were included. All other co-administrations and investigations containing multiple caffeine dosages were excluded.

In subplots Figures 6A–C, no data was excluded. In subplot Figure 6D and Figure 4, included subjects were healthy, non-smoking, non-pregnant, and non-oral contraceptive consumers. Interventions with caffeine administrated as a cocktail with negligible caffeine-drug interactions were included. All other co-administrations and investigations containing multiple caffeine dosages were excluded.

## 3 Results

### 3.1 Caffeine Pharmacokinetics Data Set

**FIGURE 2:**
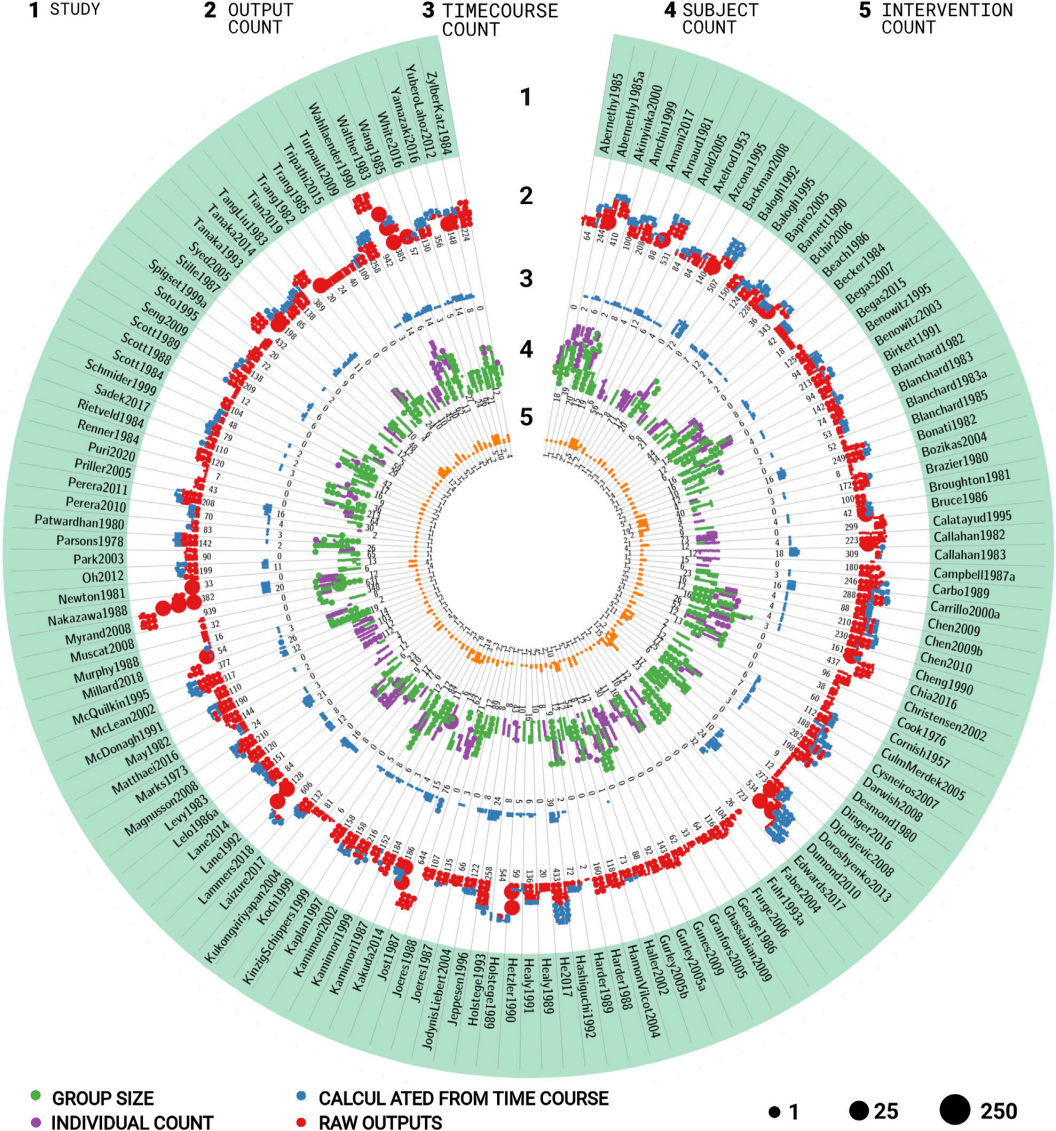
Overview of studies in the caffeine pharmacokinetics data set. The data set consists of 141 studies containing 500 groups, 4,714 individuals, 387 interventions, 24 ,571 outputs, and 846 time-courses. The circular plot is structured in stripes and rings. Each stripe represents a different study, each ring the amount of different data types for the respective study. The dots represent the respective amount of data with the dot size corresponding to the number of entries per dot. The rings contain the following information for the respective study **(A)** name of the study; **(B)** number of outputs (pharmacokinetics parameters and other measurements). Red dots represent reported data, blue dots data calculated from time-courses reported in the study; **(C)** number of time-courses; **(D)** number of participants. Purple dots represent participants with individual data, green dots represent collectively reported participants; **(E)** number of interventions applied to the participants in the study. For additional information see Table 1.

Within this work, the first comprehensive open pharmacokinetics data set on caffeine was established. The data set integrates data from 141 publications (Figure 2 and Table 1), with most of the publications corresponding to a distinct clinical trial. Studies were identified and included or excluded following the systematic approach described in the PRISMA flow diagram (Figure 1A). The focus of data curation was on pharmacokinetics data of caffeine, caffeine metabolites, and caffeine metabolic ratios in human adults. Importantly, the data set is enriched with meta-data on 1) the characteristics of studied patient cohorts and subjects (e.g., age, body weight, smoking status, health status, fasting); 2) the applied interventions (e.g., dosing, substance, route of application); 3) measured pharmacokinetic time-courses; and 4) pharmacokinetic parameters (e.g., clearance, half-life, area under the curve). In summary, data from 500 groups and 4,714 individuals is reported under 387 interventions resulting in 24 ,571 pharmacokinetic outputs and 846 time-courses. The data set is available via the pharmacokinetics database PK-DB (https://pk-db.com/) with a detailed description of the data structure provided in (Grzegorzewski et al., 2020). We demonstrate the value of the data set by its application to multiple research questions relevant for metabolic phenotyping and liver function testing (Figure 1B): 1) the effect of smoking and oral contraceptive use on caffeine elimination (Section 3.3); 2) the effect of the type of assay (Section 3.4); 3) the interaction of caffeine with other drugs (Section 3.5), 4) alteration of caffeine pharmacokinetics in disease (Section 3.6); and 5) the applicability of caffeine as a salivary test substance by comparison of plasma and saliva data (Section 3.7). In the following, we summarize the quality of reporting and provide example applications of the data set.

**TABLE 1:** Overview of curated studies. For each study the table shows which information was reported. Either, information was reported (*✓*), partially reported (⊘) or not reported at all (whitespace). The type of assay column contains reported information on quantification method for the concentration of caffeine or its metabolites (i.e., CC: Column chromatography, GC: Gas chromatography, CGC: Capillary gas chromatography, GLC: Gas-liquid chromatography, GC MS: Gas chromatography mass spectrometry, HPLC: High performance liquid chromatography, HPLC MS/MS: High performance liquid chromatography-tandem mass spectrometry, RP-HPLC: Reversed-phase high-performance liquid chromatography, LC MS Liquid chromatography mass spectrometry, LC MS/MS Liquid chromatography-tandem mass spectrometry, HPLC-ESI-MS/MS: High-performance liquid chromatography-electrospray ionisation tandem mass spectrometry, ES MS/MS: Electrospray ionisation tandem mass spectrometry, UV/Vis: UV–visible spectrophotometry, EMIT: Enzyme multiplied immunoassay technique, ELISA: enzyme-linked immunosorbent assay, RIA: Radioimmunoassay).

Study	PKDB	Type of assay	Subjects	Groups	Sex	Age	Weight	Height	BMI	Ethnicity	Healthy	Medication	Smoking	Fast	Alcohol	Dose	Route	Form	gCYP1A2	pCYP1A2	pNAT2	pXO	Caffeine	Paraxanthine	Theophylline	Theobromine	137U	17U	13U	37U	1X	1X	3X	3X	7X	7U	AFMU	AAMU	ADMU	A1U	A3U
#	141		4,931	327	52	23	21	4	2	6	56	21	40	17	2	136	139	135	0	4	0	0	69	27	11	11	0	2	0	0	2	1	0	0	0	0	1	1	0	0	0
Abernethy and Todd (1985)	PKDB00001	HPLC	18	2	*✓*	*✓*	*✓*			*✓*	*✓*	⊘	*✓*			*✓*	*✓*	*✓*					⊘																		
Abernethy et al. (1985)	PKDB00427	HPLC	39	2	*✓*	⊘	⊘		⊘		*✓*	*✓*	*✓*	*✓*		*✓*	*✓*	*✓*					*✓*																		
Akinyinka et al. (2000)	PKDB00002	HPLC	20	3	*✓*	*✓*	*✓*			*✓*	*✓*	⊘	*✓*			*✓*	*✓*	*✓*		⊘			*✓*	*✓*	⊘	⊘															
Amchin et al. (1999)	PKDB00003	HPLC	15	1	*✓*	*✓*	*✓*	*✓*			*✓*	*✓*	*✓*	*✓*		*✓*	*✓*	*✓*		⊘			*✓*	⊘		⊘		⊘			⊘	⊘						⊘			
Armani et al. (2017)	PKDB00428	HPLC MS/MS	19	1	⊘	⊘	⊘		⊘	⊘	⊘					*✓*	*✓*	*✓*					*✓*																		
Arnaud and Welsch (1982)	PKDB00429	RP-HPLC	6	1							⊘		⊘	⊘		⊘	*✓*	*✓*					*✓*	*✓*	*✓*	*✓*	⊘	⊘	⊘	⊘	⊘	⊘	⊘	⊘	⊘	⊘	⊘	⊘	⊘	⊘	⊘
Arold et al. (2005)	PKDB00430	HPLC	56	6	⊘	*✓*	*✓*	*✓*			*✓*		*✓*			*✓*	*✓*	*✓*					*✓*	⊘																	
Axelrod and Reichenthal (1953)	PKDB00431	UV/Vis	3	0	⊘											*✓*	*✓*	*✓*					*✓*																		
Azcona et al. (1995)	PKDB00432	HPLC	8	1	⊘	⊘	⊘	⊘			⊘	⊘			⊘	*✓*	*✓*	*✓*					*✓*																		
Backman et al. (2008)	PKDB00004	HPLC	71	0	⊘	⊘	⊘		⊘		⊘	⊘	⊘		⊘	*✓*	*✓*	*✓*		⊘																					
Balogh et al. (1992)	PKDB00005	HPLC	12	1	*✓*		*✓*				*✓*	*✓*	*✓*			*✓*	*✓*	*✓*					*✓*																		
Balogh et al. (1995)	PKDB00006	HPLC	20	4	*✓*						*✓*		*✓*			*✓*	*✓*	*✓*					⊘																		
Bapiro et al. (2005)	PKDB00433	HPLC	10	3	*✓*						*✓*		*✓*			*✓*	*✓*	*✓*		⊘			*✓*	⊘																	
Barnett et al. (1990)	PKDB00007	HPLC	6	0	⊘	⊘	⊘				⊘	⊘	⊘		⊘	*✓*	*✓*	*✓*					⊘																		
Bchir et al. (2006)	PKDB00434	HPLC	8	2	⊘	⊘	⊘		⊘		⊘			⊘		*✓*	*✓*	*✓*					*✓*																		
Beach et al. (1986)	PKDB00008	HPLC	21	4	*✓*						*✓*	*✓*	*✓*	*✓*		*✓*	*✓*	*✓*					⊘																		
Becker et al. (1984)	PKDB00435	HPLC	23	2	⊘						⊘	⊘				*✓*	*✓*	*✓*					*✓*		*✓*																
Begas et al. (2007)	PKDB00009	HPLC	44	5	⊘	⊘					⊘	⊘	⊘			*✓*	*✓*	*✓*		⊘	⊘	⊘																			
Begas et al. (2015)	PKDB00436	RP-HPLC	34	2	*✓*						*✓*	*✓*	⊘			*✓*	*✓*	*✓*		⊘			⊘	⊘				⊘			⊘	⊘					⊘				
Benowitz et al. (1995)	PKDB00010	HPLC	12	2	⊘	⊘					⊘	⊘	⊘			*✓*	*✓*	*✓*		⊘			*✓*	*✓*																	
Benowitz et al. (2003)	PKDB00011	HPLC	12	3	*✓*						*✓*		*✓*	*✓*		*✓*	*✓*	*✓*			⊘	⊘	*✓*																		
Birkett and Miners (1991)	PKDB00012	HPLC	6	0	⊘	⊘	⊘				⊘		⊘			*✓*	*✓*	*✓*					⊘																		
Blanchard and Sawers (1983b)	PKDB00127	HPLC	16	2	⊘	⊘	⊘	⊘		⊘	⊘		⊘		⊘	*✓*	*✓*	*✓*					*✓*																		
Blanchard and Sawers (1983c)	PKDB00210	-	16	2	*✓*	*✓*					*✓*			*✓*	*✓*	*✓*	*✓*	*✓*					⊘																		
Blanchard and Sawers (1983a)	PKDB00013	HPLC	10	1	⊘	⊘	⊘			⊘	⊘	⊘	⊘	⊘	⊘	*✓*	*✓*	*✓*					*✓*																		
Blanchard et al. (1985)	PKDB00378	HPLC	12	2	⊘	⊘	⊘			⊘	⊘	⊘	⊘	⊘		*✓*	*✓*	*✓*					⊘	⊘	⊘	⊘	⊘	⊘	⊘		⊘	⊘	⊘		⊘	⊘					
Bonati et al. (1982)	PKDB00014	HPLC	4	1	*✓*		*✓*				*✓*			*✓*		*✓*	*✓*	*✓*					*✓*	*✓*	*✓*	*✓*		⊘			⊘		⊘		⊘				⊘		
Bozikas et al. (2004)	PKDB00437	HPLC	40	4	⊘	⊘					⊘		⊘			*✓*	*✓*	*✓*		⊘																					
Brazier et al. (1980)	PKDB00438	GC	4	0	⊘						⊘					*✓*	*✓*	*✓*					*✓*	⊘																	
Broughton and Rogers (1981)	PKDB00439	GLC	5	0	⊘						⊘	⊘	⊘	⊘		*✓*	*✓*	*✓*					⊘																		
Bruce et al. (1986)	PKDB00440	HPLC	9	1	⊘						⊘				⊘	*✓*	*✓*	*✓*					*✓*	*✓*																	
Calatayud et al. (1995)	PKDB00441	EMIT	73	2	*✓*	⊘					*✓*	*✓*	*✓*			*✓*	*✓*	*✓*					⊘																		
Callahan et al. (1982)	PKDB00442	HPLC	12	5			⊘				*✓*		*✓*	*✓*		*✓*	*✓*	*✓*					*✓*	⊘	⊘	⊘	⊘	⊘	⊘		⊘	⊘	⊘		⊘						
Callahan et al. (1983)	PKDB00443	HPLC	12	3	*✓*		*✓*				*✓*		*✓*	*✓*		*✓*	*✓*	*✓*					*✓*	*✓*		*✓*					*✓*	*✓*						*✓*			
Campbell et al. (1987)	PKDB00015	HPLC	15	3	*✓*	*✓*	*✓*			*✓*	*✓*		*✓*			*✓*	*✓*	*✓*					⊘					⊘			⊘	⊘					⊘				
Carbó et al. (1989)	PKDB00016	HPLC	6	1	*✓*	*✓*	*✓*				*✓*	*✓*	*✓*		*✓*	*✓*	*✓*	*✓*		⊘			⊘																		
Carrillo et al. (2000)	PKDB00017	HPLC	23	4	⊘	⊘	⊘				⊘	⊘	⊘			*✓*	*✓*	*✓*		⊘			⊘					*✓*													
Chen et al. (2009a)	PKDB00445	HPLC	16	1	⊘	⊘	⊘	⊘		⊘	⊘	⊘	⊘	⊘		*✓*	*✓*	*✓*		⊘			*✓*	*✓*																	
Chen et al. (2009b)	PKDB00446	HPLC	12	1	*✓*					*✓*	*✓*	*✓*	*✓*	*✓*		*✓*	*✓*	*✓*			⊘	⊘	*✓*	*✓*				⊘			⊘	⊘					⊘				
Chen et al. (2010)	PKDB00447	HPLC	16	1	*✓*	*✓*	*✓*	*✓*		*✓*	*✓*	*✓*	*✓*	*✓*		*✓*	*✓*	*✓*			⊘	⊘	*✓*	*✓*				⊘			⊘	⊘					⊘				
Cheng et al. (1990)	PKDB00018	HPLC	26	3	*✓*	⊘	⊘				*✓*		*✓*			*✓*	*✓*	*✓*					⊘																		
Chia et al. (2016)	PKDB00448	HPLC	52	12	*✓*					*✓*	*✓*		*✓*			*✓*	*✓*	*✓*		⊘			⊘	⊘																	
Christensen et al. (2002)	PKDB00449	HPLC	12	0	⊘	⊘	⊘				⊘	⊘	⊘			*✓*	*✓*	*✓*					⊘																		
Cook et al. (1976)	PKDB00450	RIA	13	0												*✓*	*✓*	*✓*					⊘																		
Cornish and Christman (1957)	PKDB00128	CC	2	0	⊘						⊘					*✓*	*✓*						⊘	⊘	⊘	⊘			⊘		⊘	⊘	⊘		⊘	⊘					
Culm-Merdek et al. (2005)	PKDB00019	HPLC	7	1	⊘	⊘	⊘				⊘		⊘			*✓*	*✓*	*✓*					*✓*	*✓*	⊘	⊘															
Cysneiros et al. (2007)	PKDB00451	RP-HPLC	12	1	⊘						⊘	⊘	⊘			*✓*	*✓*	*✓*					*✓*	⊘	⊘	⊘															
Darwish et al. (2008)	PKDB00328	HPLC	77	3	⊘	⊘			⊘	⊘	⊘	⊘	⊘	⊘		*✓*	*✓*	*✓*					*✓*																		
Desmond et al. (1980)	PKDB00384	HPLC	25	2	*✓*	⊘	⊘				*✓*	⊘	⊘	*✓*		*✓*	*✓*	*✓*					⊘																		
Dinger et al. (2016)	PKDB00452	LC MS/MS	6	1	⊘											*✓*	*✓*	*✓*					⊘																		
Djordjevic et al. (2008)	PKDB00453	RP-HPLC	249	12	⊘	⊘	⊘			⊘	⊘		⊘			*✓*	*✓*			⊘																					
Doroshyenko et al. (2013)	PKDB00138	LC MS	17	1	⊘	⊘			⊘		⊘		⊘		⊘	*✓*	*✓*	*✓*					⊘																		
Dumond et al. (2010)	PKDB00499	LC MS	23	1	⊘	⊘	⊘	⊘	⊘	⊘	⊘					*✓*	*✓*	*✓*			⊘	⊘	*✓*																		
Edwards et al. (2017)	PKDB00496	HPLC MS/MS	236	8	⊘	⊘	⊘	⊘	⊘		⊘	⊘		⊘		*✓*	*✓*	*✓*					*✓*																		
Faber and Fuhr (2004)	PKDB00032	HPLC	12	2	*✓*	*✓*	*✓*		*✓*		*✓*	*✓*	*✓*			*✓*	*✓*	*✓*					⊘																		
Fuhr et al. (1993)	PKDB00033	HPLC	12	2	*✓*	*✓*	*✓*	*✓*			*✓*		*✓*			*✓*	*✓*	*✓*					⊘																		
Furge and Fletke (2007)	PKDB00454	RP-HPLC	10	1									*✓*			*✓*	*✓*	*✓*		⊘			⊘	⊘																	
George et al. (1986)	PKDB00034	HPLC	10	4	*✓*						*✓*	*✓*	*✓*		⊘	*✓*	*✓*	*✓*					⊘																		
Ghassabian et al. (2009)	PKDB00035	ES MS/MS	11	0	⊘	⊘					⊘	⊘				*✓*	*✓*	*✓*		⊘																					
Granfors et al. (2005)	PKDB00036	HPLC	30	2	*✓*	*✓*	*✓*				*✓*		*✓*			*✓*	*✓*	*✓*		⊘																					
Gunes et al. (2009)	PKDB00455	HPLC	146	0	⊘						⊘	⊘	⊘			*✓*	*✓*	*✓*	⊘	⊘																					
Gurley et al. (2005a)	PKDB00456	HPLC	12	1	*✓*	*✓*	*✓*				*✓*	*✓*	*✓*			*✓*	*✓*	*✓*		⊘																					
Gurley et al. (2005b)	PKDB00457	HPLC	12	1	*✓*	*✓*	*✓*				*✓*	*✓*	*✓*			*✓*	*✓*	*✓*		⊘																					
Haller et al. (2002)	PKDB00037	LC MS/MS	8	1	⊘		*✓*				*✓*		*✓*			*✓*	*✓*	*✓*					*✓*																		
Hamon-Vilcot et al. (2004)	PKDB00458	HPLC	54	2	*✓*	*✓*			*✓*		*✓*	*✓*				*✓*	*✓*	*✓*		⊘																					
Harder et al. (1988)	PKDB00038	HPLC	12	1	⊘		⊘				⊘	⊘	⊘			*✓*	*✓*	*✓*					⊘	⊘																	
Harder et al. (1989)	PKDB00039	HPLC	12	1	⊘		⊘				⊘	⊘	⊘			*✓*	*✓*	*✓*					⊘																		
Hashiguchi et al. (1992)	PKDB00459	HPLC	9	1	*✓*	*✓*					*✓*		*✓*			*✓*	*✓*	*✓*					*✓*																		
He et al. (2017)	PKDB00460	LC MS/MS	8	1	*✓*						*✓*	*✓*	*✓*			*✓*	*✓*	*✓*					*✓*	⊘																	
Healy et al. (1989)	PKDB00040	RP-HPLC	10	1	⊘	⊘	⊘				⊘		⊘			*✓*	*✓*	*✓*					⊘	⊘																	
Healy et al. (1991)	PKDB00041	RP-HPLC	16	1	⊘	⊘	⊘				⊘	⊘	⊘	⊘		*✓*	*✓*	*✓*		⊘			*✓*	*✓*																	
Hetzler et al. (1990)	PKDB00042	RP-HPLC	10	1	⊘	⊘	⊘	⊘			⊘			⊘		*✓*	*✓*	*✓*		*✓*			*✓*	*✓*																	
Holstege et al. (1989)	PKDB00461	HPLC	43	6	⊘	⊘					*✓*	*✓*				*✓*	*✓*	*✓*					*✓*	*✓*	*✓*	*✓*															
Holstege et al. (1993)	PKDB00462	HPLC	8	1	*✓*	⊘	⊘				*✓*		*✓*			*✓*	*✓*	*✓*					*✓*	*✓*	*✓*	*✓*															
Jeppesen et al. (1996)	PKDB00043	HPLC	8	1	⊘						⊘	⊘	⊘			*✓*	*✓*	*✓*					⊘	⊘	*✓*	⊘															
Jodynis-Liebert et al. (2004)	PKDB00382	HPLC	89	6	⊘	⊘	⊘				⊘	⊘	⊘			*✓*	*✓*	*✓*		⊘			⊘																		
Joeres et al. (1987)	PKDB00385	GC	12	2	*✓*						*✓*	⊘		*✓*		*✓*	*✓*	*✓*					*✓*																		
Joeres et al. (1988)	PKDB00044	GC	71	5	*✓*	⊘	⊘				*✓*	⊘	*✓*			*✓*	*✓*	*✓*					⊘																		
Jost et al. (1987)	PKDB00463	EMIT	62	0	⊘	⊘	⊘				⊘		⊘			*✓*	*✓*	*✓*					*✓*																		
Kakuda et al. (2014)	PKDB00137	LC MS/MS	14	1	⊘	⊘			⊘	⊘	⊘	⊘	⊘		⊘	*✓*	*✓*	*✓*					*✓*																		
Kamimori et al. (1987)	PKDB00464	HPLC	6	2	*✓*	*✓*	*✓*				*✓*		*✓*	*✓*			⊘	⊘					*✓*																		
Kamimori et al. (1999)	PKDB00465	HPLC	11	3	*✓*	*✓*	*✓*				*✓*		*✓*	*✓*		*✓*	*✓*	*✓*					*✓*																		
Kamimori et al. (2002)	PKDB00468	HPLC	84	1	⊘						⊘	⊘	⊘		⊘	*✓*	*✓*	*✓*					*✓*																		
Kaplan et al. (1997)	PKDB00045	HPLC	12	1	*✓*	*✓*	*✓*				*✓*		*✓*			*✓*	*✓*	*✓*					*✓*	*✓*	⊘	*✓*															
Kinzig-Schippers et al. (1999)	PKDB00467	HPLC	12	1	⊘	⊘	⊘				⊘	⊘	⊘			*✓*	*✓*	*✓*					*✓*																		
Koch et al. (1999)	PKDB00046	RP-HPLC	6	0		⊘	⊘				⊘					*✓*	*✓*	*✓*		⊘																					
Kukongviriyapan et al. (2004)	PKDB00469	HPLC	77	9	*✓*	*✓*	⊘			⊘	⊘		*✓*	⊘		*✓*	*✓*	*✓*		⊘			⊘																		
Laizure et al. (2017)	PKDB00470	HPLC-ESI-MS/MS	17	3	⊘	⊘	⊘				⊘	⊘	⊘			*✓*	*✓*	*✓*					⊘	*✓*	*✓*	*✓*															
Lammers et al. (2018)	PKDB00471	LC MS/MS	9	2	*✓*						*✓*			⊘		*✓*	*✓*	*✓*					*✓*																		
Lane et al. (1992)	PKDB00472	RIA	10	2	*✓*	⊘					*✓*					*✓*	*✓*	*✓*					⊘																		
Lane et al. (2014)	PKDB00341	HPLC	23	2	⊘	⊘				⊘	⊘	⊘	⊘			*✓*	*✓*	*✓*					*✓*	*✓*																	
Lelo et al. (1986a)	PKDB00047	HPLC	6	1	*✓*						*✓*	*✓*	*✓*	*✓*		*✓*	*✓*	*✓*					*✓*	*✓*	*✓*	*✓*															
Levy and Zylber-Katz (1983)	PKDB00048	HPLC	12	0	⊘	⊘	⊘				⊘	⊘	⊘			*✓*	*✓*	*✓*					⊘																		
Magnusson et al. (2008)	PKDB00049	HPLC MS/MS	7	0	⊘					⊘	⊘	⊘	⊘			*✓*	*✓*	*✓*		⊘			⊘	⊘																	
Marks and Kelly (1973)	PKDB00338	UV/Vis	3	1	⊘									⊘		*✓*	*✓*	*✓*					*✓*																		
Matthaei et al. (2016)	PKDB00473	HPLC	112	12	*✓*	⊘	⊘	⊘	⊘		*✓*		⊘			*✓*	*✓*		⊘	⊘			⊘																		
May et al. (1982)	PKDB00050	HPLC	12	2	*✓*						*✓*	*✓*	*✓*	*✓*		*✓*	*✓*	*✓*					⊘																		
McDonagh et al. (1991)	PKDB00474	EMIT	103	8		⊘					⊘					*✓*	*✓*	*✓*					⊘																		
McLean and Graham (2002)	PKDB00051	HPLC	14	2	⊘	⊘	⊘				⊘	⊘	⊘		⊘	*✓*	*✓*	*✓*					*✓*	*✓*	*✓*	*✓*															
McQuilkin et al. (1995)	PKDB00475	RP-HPLC	19	0	⊘	⊘				⊘	⊘		⊘	⊘		*✓*	*✓*	*✓*		*✓*			*✓*	*✓*				*✓*			*✓*						*✓*				
Millard et al. (2018)	PKDB00381	GC-MS,ELISA	2	0												*✓*	*✓*	*✓*	⊘				*✓*																		
Murphy et al. (1988)	PKDB00052	HPLC	8	4	⊘	⊘	⊘				⊘	⊘	⊘			*✓*	*✓*	*✓*					⊘																		
Muscat et al. (2008)	PKDB00476	HPLC	348	20	⊘					⊘	⊘		⊘				*✓*	*✓*		⊘																					
Myrand et al. (2008)	PKDB00497	HPLC	631	0	⊘	⊘	⊘	⊘		⊘	⊘		⊘			*✓*	*✓*	*✓*	⊘	⊘																					
Nakazawa and Tanaka (1988)	PKDB00477	HPLC	12	1	⊘	⊘	⊘				*✓*					*✓*	*✓*						*✓*	*✓*	*✓*	*✓*															
Newton et al. (1981)	PKDB00053	GLC	6	1	⊘	⊘	⊘				⊘		⊘	⊘		*✓*	*✓*	*✓*					⊘																		
Oh et al. (2012)	PKDB00054	LC MS/MS	13	1							⊘					*✓*	*✓*	*✓*		⊘			*✓*	*✓*																	
Park et al. (2003)	PKDB00055	GC	65	0	⊘	⊘	⊘				⊘	⊘	⊘			*✓*	*✓*	*✓*					⊘																		
Parsons and Neims (1978)	PKDB00056	RIA	26	2	*✓*	*✓*					*✓*	*✓*	⊘			*✓*	*✓*	*✓*					*✓*																		
Patwardhan et al. (1980)	PKDB00057	HPLC	31	3	⊘		⊘				⊘	⊘	⊘	⊘	⊘	*✓*	*✓*	*✓*					*✓*																		
Perera et al. (2010)	PKDB00478	HPLC	2	0	⊘	⊘	⊘				⊘		⊘			*✓*	*✓*	*✓*		⊘			*✓*	*✓*																	
Perera et al. (2011)	PKDB00058	HPLC	30	2	⊘						⊘	⊘	⊘			*✓*	*✓*	*✓*		⊘			*✓*	⊘																	
Priller (2005)	PKDB00479	HPLC	64	11	*✓*	⊘			⊘		*✓*		⊘			*✓*	*✓*	*✓*		⊘																					
Puri et al. (2020)	PKDB00480	Spectrophotometry	213	2	*✓*	*✓*					*✓*		*✓*			*✓*	*✓*	*✓*					⊘																		
Renner et al. (1984)	PKDB00059	GLC	36	4	*✓*	*✓*	*✓*				*✓*		*✓*			*✓*	*✓*	*✓*					⊘																		
Rietveld et al. (1984)	PKDB00481	GLC	9	0	⊘	⊘	⊘				⊘		⊘			*✓*	*✓*	*✓*					*✓*																		
Sadek et al. (2017)	PKDB00482	LC MS/MS	16	2							⊘	⊘	⊘			*✓*	*✓*	*✓*					⊘																		
Schmider et al. (1999)	PKDB00483	HPLC	17	1	⊘						⊘	⊘	⊘			*✓*	*✓*	*✓*					*✓*																		
Scott et al. (1984)	PKDB00484	RP-HPLC	42	0																			⊘																		
Scott et al. (1988)	PKDB00485	HPLC	24	3	⊘	⊘	⊘				⊘	⊘	⊘	⊘		*✓*	*✓*	*✓*					⊘																		
Scott et al. (1989)	PKDB00486	RP-HPLC	29	3	*✓*	⊘	⊘				*✓*	⊘				*✓*	*✓*	*✓*					*✓*	*✓*																	
Seng et al. (2009)	PKDB00060	HPLC	59	3	⊘	⊘	⊘			⊘	⊘		⊘			*✓*	*✓*	*✓*					⊘																		
Soto et al. (1995)	PKDB00487	HPLC	5	1	*✓*	*✓*	*✓*				*✓*	*✓*	*✓*			*✓*	*✓*	*✓*					⊘																		
Spigset et al. (1999)	PKDB00061	HPLC	12	1	⊘	⊘	⊘				⊘	⊘	⊘	⊘		*✓*	*✓*	*✓*		*✓*			⊘																		
Stille et al. (1987)	PKDB00062	HPLC	12	0	⊘		⊘				⊘	⊘	⊘			*✓*	*✓*	*✓*					⊘																		
Syed et al. (2005)	PKDB00488	HPLC	48	3	⊘						⊘	⊘	⊘			*✓*	*✓*	*✓*					*✓*																		
Tanaka et al. (1993)	PKDB00489	GC	10	1	⊘						⊘	⊘		⊘		*✓*	*✓*	*✓*					*✓*	*✓*	*✓*	*✓*															
Tanaka et al. (2014)	PKDB00136	HPLC-ESI-MS/MS	4	1	⊘						⊘					*✓*	*✓*	*✓*		*✓*			*✓*	*✓*																	
Tang-Liu et al. (1983)	PKDB00126	RP-HPLC	6	1	*✓*		⊘				*✓*	*✓*		*✓*		*✓*	*✓*	*✓*					⊘	⊘	⊘	⊘	⊘	⊘	⊘		⊘	⊘	⊘		⊘	⊘					
Tian et al. (2019)	PKDB00490	HPLC	24	2	⊘					⊘	⊘		⊘			*✓*	*✓*	*✓*	⊘	⊘																					
Trang et al. (1982)	PKDB00129	HPLC	10	1	⊘	⊘	⊘	⊘			⊘					*✓*	*✓*	*✓*					⊘																		
Trang et al. (1985)	PKDB00491	HPLC	10	0	⊘						⊘	⊘	⊘			*✓*	*✓*	*✓*					⊘																		
Tripathi et al. (2015)	PKDB00492	EMIT	45	5	*✓*	⊘					*✓*					*✓*	*✓*	*✓*					*✓*																		
Turpault et al. (2009)	PKDB00063	LC MS/MS	30	1	⊘						⊘	⊘	⊘	⊘		*✓*	*✓*	*✓*		⊘			⊘	⊘																	
Wahlländer et al. (1990)	PKDB00493	EMIT,RP-HPLC	62	10	*✓*	⊘					*✓*		⊘			*✓*	*✓*	*✓*					⊘																		
Walther et al. (1983)	PKDB00500	GC	13	4	*✓*	⊘	⊘				*✓*					*✓*	*✓*	*✓*					*✓*																		
Wang et al. (1985)	PKDB00383	CGC	27	3	⊘	⊘					⊘	⊘	⊘	⊘		*✓*	*✓*	*✓*					*✓*																		
White et al. (2016)	PKDB00339	LC MS	24	1	⊘	⊘	⊘	⊘	⊘		⊘	⊘	⊘	⊘		*✓*	*✓*	*✓*					*✓*																		
Yamazaki et al. (2017)	PKDB00494	LC MS	96	6	⊘	⊘	⊘		⊘	⊘	⊘	⊘				*✓*	*✓*	*✓*					*✓*																		
Yubero-Lahoz et al. (2012)	PKDB00495	HPLC	21	2	⊘	⊘	⊘	⊘		⊘	⊘		⊘	⊘		*✓*	*✓*	*✓*					*✓*	*✓*																	
Zylber-Katz et al. (1984)	PKDB00065	HPLC	12	0	⊘	⊘	⊘				⊘	⊘	⊘			⊘	*✓*	*✓*					⊘																		

### 3.2 Reporting of Pharmacokinetics Data

The study design as well as quality and details of reporting of results were very heterogeneous between studies. Major differences exist in the study design, number of study participants, and number of reported time-courses. Many studies report some individual participants data (84/141) but only a minority of the studies report individual participant data for all study participants (6/84) (Axelrod and Reichenthal, 1953; Brazier et al., 1980; Rietveld et al., 1984; McQuilkin et al., 1995; Perera et al., 2010; Millard et al., 2018). Many studies report only aggregated data on group level (57/141). In most studies, the application of a single dose of caffeine was studied (129/141). In the case of multiple interventions (49/141), mostly one additional substance was co-administrated (33/141). The main categories of studies were either 1) case-control studies which compare caffeine pharmacokinetics in two groups (e.g., healthy vs Disease) (64/141), 2) crossover studies on caffeine-drug interactions (comparing caffeine alone vs caffeine and additional substance) (33/141) 3) studies on metabolic phenotypes (including drug cocktails) (42/141); or 4) methodological studies (e.g., establishing mass spectrometry protocol for quantification or new site of sampling).

Intervention protocols, i.e., the applied substances, form, dose, and timing of application was typically reported in good detail. In crossover studies, the difference between treatments was generally reported in good detail. For the dosing with caffeine, the amount (136/141), route (e.g., oral, intravenous) (140/141), form (tablet, capsule, solution) (136/141), and the substance (141/141) are typically reported. Co-administrations of medication and other substances are often mentioned qualitatively (28/49), skipping either the amount, route, form, or exact timing of application.

The quantification protocol, i.e., type of assay (e.g., high-performance liquid chromatography, gas-liquid chromatography) (140/141), the site of sampling (e.g., plasma, serum, saliva, urine) (141/141), and the time points when samples were taken (128/141), were mostly reported in good detail. However, in some studies the protocol is not mentioned explicitly but only via additional references, which complicates the curation. For a single study no information on the quantification method could be accessed (Blanchard and Sawers, 1983c). The reporting of type of assay is very heterogeneous with many studies not providing sufficient details (e.g., HPLC is often mentioned as the separation technique but the subsequent detection or quantification method is not stated).

The information on subject characteristics was less often reported in sufficient detail with large differences in the quality and quantity of reporting between studies. Any information on sex (131/141), weight (71/141), and age (89/141) was relatively often reported on group or individual level. However, age and weight were rarely reported on an individual level and often not even for all groups. Other anthropometric factors such as height (15/141), body mass index (BMI) (15/141), and ethnicity (25/141) are rarely reported. The genotype of CYP1A2 (gCYP1A2) is rarely measured or reported (5/141), even though there is evidence that genetic variation can play an important role in caffeine metabolism. Further, the nomenclature is not standardized. It is worth noting that low-cost genotyping methods were not available for early studies included in the data set. The phenotype (pCYP1A2, pXO, pNAT2) of enzymes involved in the metabolism of caffeine, i.e., CYP1A2, xanthine oxidase (XO), or N-acetyltransferase type 2 (NAT2) were investigated occasionally (38/141). The information on other factors influencing the pharmacokinetics of caffeine is reported very heterogeneously. The strong influence of smoking (105/141) and the use of oral contraceptives (42/141) on the enzyme activity of CYP1A2 and thereby on the apparent clearance of caffeine is covered relatively well in many publications. Health status and patient diseases are often covered (134/141). However, often categorized in broad and general disease classes, with more specific disease classification lacking. Markers related to cardiovascular health (e.g., blood pressure, cholesterol level, bilirubin level) are basically not reported in the context of caffeine pharmacokinetics. In case of cirrhosis, further information of severity is reported sparsely. Important information on the abstinence of caffeine or methylxanthines and consumption of caffeine or other caffeine-containing beverages is often missing.

Individual-level reporting is essential for subsequent pharmacokinetic modeling, as biological mechanisms responsible for the pharmacokinetics strongly correlate with these factors and large inter-individual variability exists in caffeine pharmacokinetics. Despite the importance of individual subject data, information on individuals is rarely provided.

Most studies report pharmacokinetics outputs on caffeine (126/141). Data on the main product paraxanthine (46/141) and secondary metabolites theobromine (20/141) and theophylline (19/141) are reported sometimes. Additional metabolites such as 137U, 17U, 13U, 37U, 1X, 1U, 3X, 3U, 7X, 7U, AFMU, AAMU, ADMU, A1U, and A3U are seldom reported, mostly as urinary measurements and not directly but as part of a metabolic ratio.

### 3.3 Smoking and Oral Contraceptives

**FIGURE 3:**
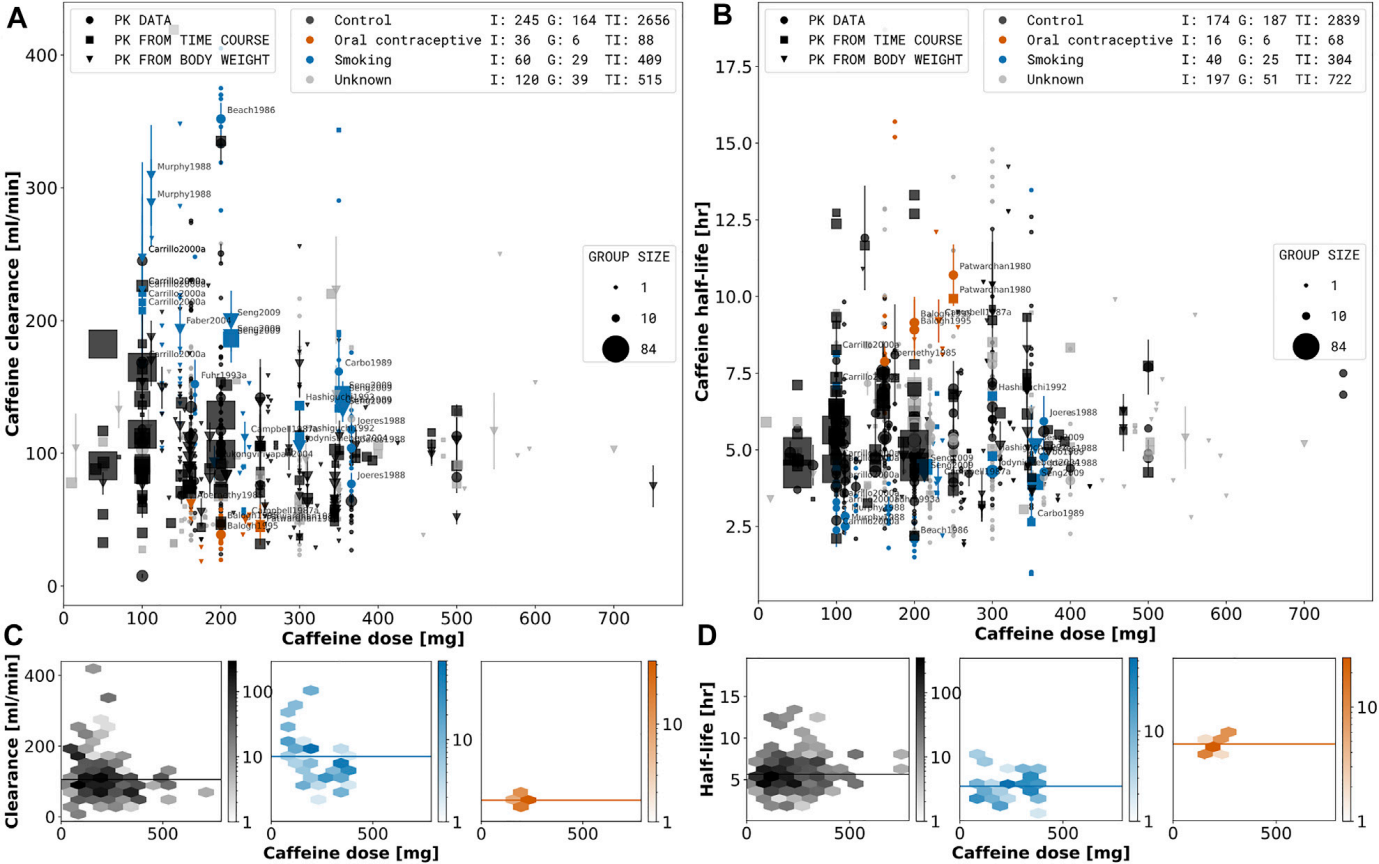
Dose-dependent effect of smoking and oral contraceptive use on caffeine pharmacokinetics. A stratified meta-analysis of caffeine clearance **(A)** and half-life **(B)** depending on reported smoking and oral contraceptive use and dose was performed. Black: Control subjects are non-smokers and not taking oral contraceptives; Orange: Oral contraceptive users independent of smoking status (smokers and non-smokers). Blue: Smoking are smokers not consuming oral contraceptives. Grey: Unknown data correspond to subjects with unreported smoking and oral contraceptive status. Marker shape, and size describe the datatype and group size, respectively. Data representing smokers or oral contraceptive consumers is labeled by the respective study name. The hexagonal bin plots in the lower panel **(C, D)** correspond to the subset of data for the control, smoking, and oral contraceptive consuming subjects. The color intensity of each bin represents the number of subjects falling in a given hexagonal bin area. Data selection criteria and visualization are described in the Section 2.

In a first analysis, we were interested in the effect of smoking and oral contraceptive use on the pharmacokinetics of caffeine in healthy subjects (see Figure 3). Both have repeatedly been reported as key exogenous factors affecting caffeine elimination. A main question was how reproducible the effect is and if by integrating data from multiple studies a more consistent picture of the effects can be gained. For the analysis the data set was stratified into smokers, oral contraceptive users and a control group (neither smoking nor using oral contraceptives). Smoking results in increased caffeine clearance (Figures 3A,C) and decreased half-life of caffeine elimination (Figures 3B,D) whereas oral contraceptive use has the opposite effect over a wide dose range of caffeine. An important result from our analysis is that a consistent and reproducible effect can be found over more than 50 years of pharmacokinetic research. With exception of a few outlier studies probably from a single clinical trial (see methods) all data was highly consistent. This provides a strong argument for the applied methods and protocols.

Despite the large effect of smoking and oral contraceptive use on the pharmacokinetics of caffeine, the information is only reported for a subset of studies. Smoking as well as oral contraceptive use should be an exclusion criteria for subjects in studies of caffeine pharmacokinetics, due to the possible confounding effects. Importantly, in many groups smokers and non-smokers were mixed without reporting data for smokers and non-smokers separately. Without reporting of data on individuals or subgroups no stratification could be performed, which could strongly affect results if not balanced between groups. In summary, the integrative data analysis showed a consistent strong activating effect of smoking on caffeine elimination and an inhibiting effect of oral contraceptive use on caffeine elimination.

### 3.4 Effect of Type of Assay

**FIGURE 4:**
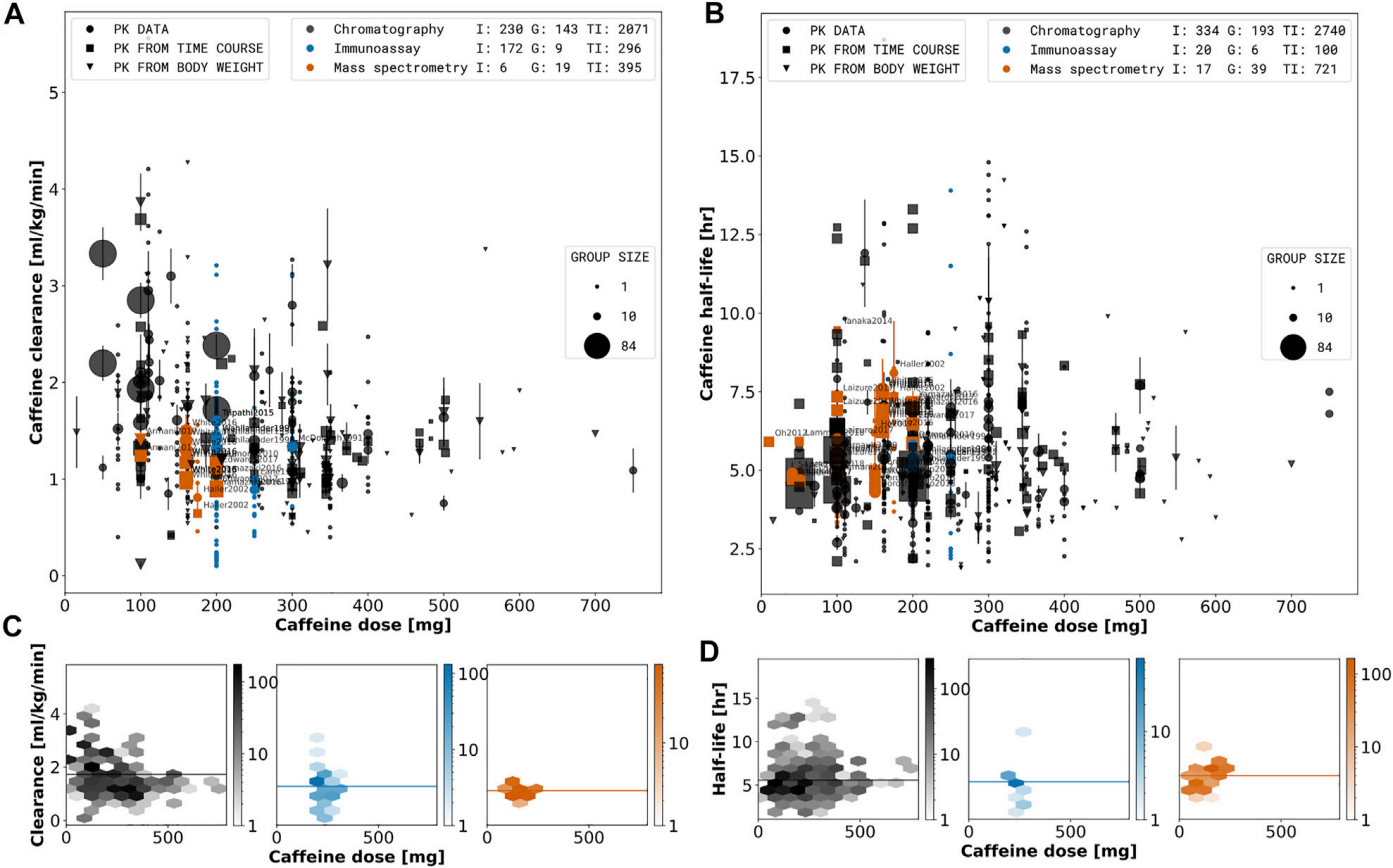
Effect of type of assay on pharmacokinetic parameters. A stratified meta-analysis of caffeine clearance **(A)** and half-life **(B)** depending on reported assay type and dose. Orange: Measurements performed with mass spectrometry (LC MS, LC MS/MS, HPLC MS/MS, HPLC-ESI-MS/MS, ES MS/MS). Blue: Measurements performed with immunoassay (RIA, EMIT). Black: Separation performed with chromatography but quantification assay was not reported (HPLC, RP-HPLC, GLC, CGC, CC, GC). Marker shape, and size describe the datatype and group size, respectively. Data with reported quantification method are labeled by the respective study name. The hexagonal bin plots in the lower panel **(C, D)** correspond to the subset of data for the mass spectrometry quantification, immunoassay quantification and chromatography. The color intensity of each bin represents the number of subjects falling in a given hexagonal bin area. Data selection criteria and visualization are described in the Section 2.

The reported type of assays were curated systematically (see Table 1 to study effects of the type of technique and assay on the reported pharmacokinetics data (see Figure 4, abbreviations explained in the legend of Table 1). Data affected by confounding factors (i.e., smoking, oral contraceptive use, caffeine-disease, and caffeine-disease interactions) was excluded. Based on our analysis no systematic difference between the reported type of assays could be detected. Immunoassays (RIA, EMIT, ELISA), mass spectrometry (LC MS, GC MS, LC MS/MS, HPLC MS/MS, HPLC-ESI-MS/MS, ES MS/MS), and chromatography with unspecified quantification method (HPLC, RP-HPLC, GLC, CGC, CC, GC) do not show any differences in half-life or clearance. Spectrophotometric methods (UV/Vis) are generally rare and were not applied in this data subset. Based on the analysis we conclude that no systematic correction was required to compare the data collected with the different types of assays.

### 3.5 Caffeine-Drug Interactions

**FIGURE 5:**
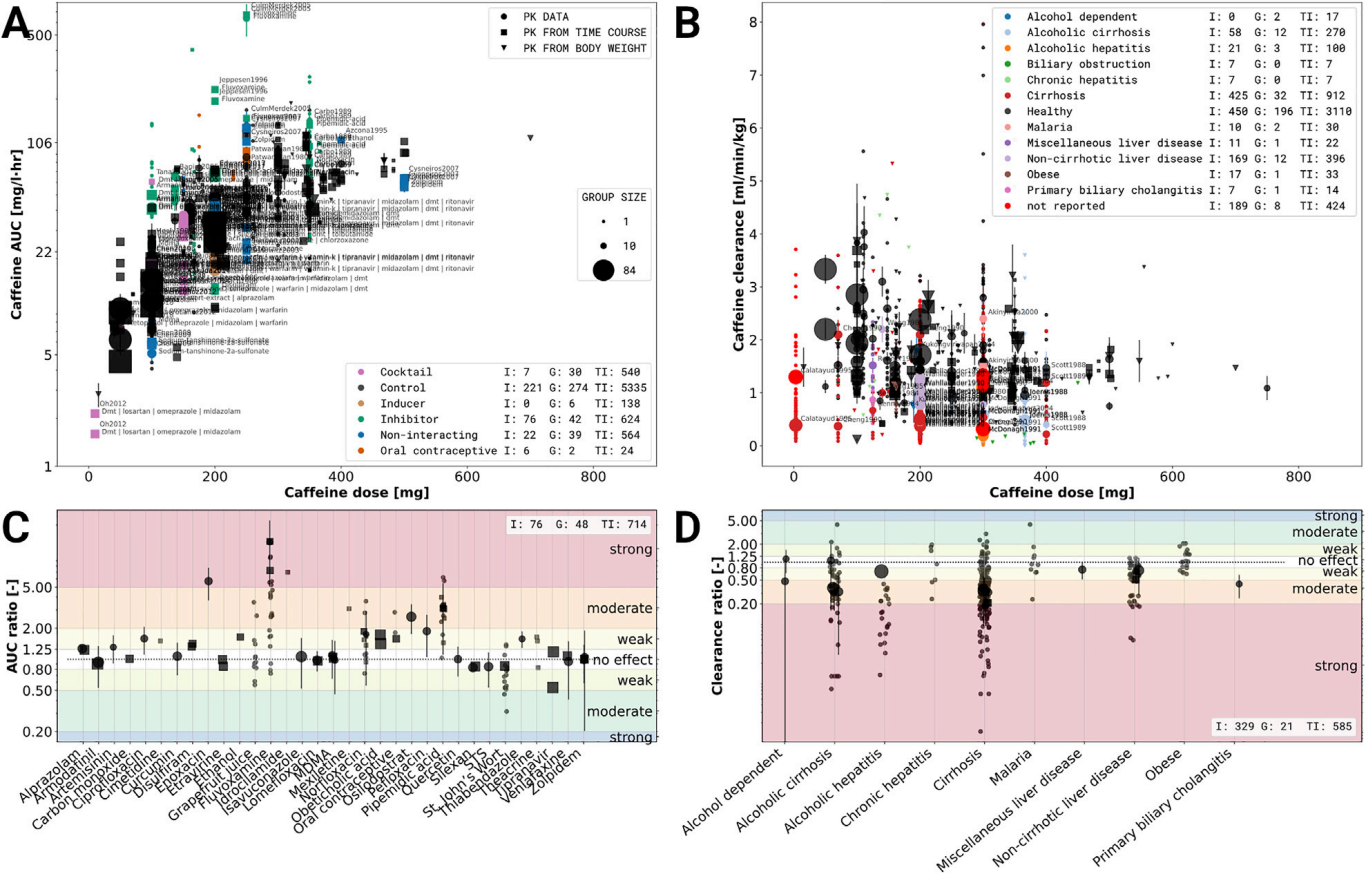
Effects of caffeine-drug and caffeine-disease interactions. **(A)** Caffeine-drug interactions based on the caffeine area under the concentration curve (AUC). Data is stratified based on co-administration of drugs with caffeine and dose. Violet: caffeine administrated as part of a drug cocktail. Common co-administrations are dextrometorphan, metoprolol, midazolam, omeprazole, and warfarin.; Black: single caffeine administration (no co-administration); Brown: co-administration with an inducing effect on the elimination of caffeine; Green: co-administration with an inhibiting effect on the elimination of caffeine; Blue: co-administrations with no effect on the pharmacokinetics of caffeine; Orange co-administration of oral contraceptives. **(B)** Caffeine-disease interactions based on caffeine clearance and dose. Data was stratified based on the health status and reported diseases, with black data points corresponding to healthy subjects. **(C)** Effect sizes of caffeine-drug interaction for studies with a controlled study design, mostly randomized control trials (RCT). The effect size is based on the log AUC ratio between caffeine application alone and caffeine with co-administration of the respective drug. The drugs were characterized as having either a strong, moderate, weak or no effect. Strong, moderate and weak inhibitors increase the AUC ≥5 -fold, ≥2 to <5 -fold, ≥1.25 to <2 -fold, respectively. **(D)** Effect size of caffeine-disease interactions for studies with a controlled study design, mostly case-controlled studies. The effect size is based on the log clearance ratio between subjects with and without a specific condition/disease. The diseases were characterized as having either a strong, moderate, weak or no effect. Strong, moderate and weak effect decreased the clearance by ≥80 percent, ≥50 to <80 percent and ≥20 to <50 percent, respectively. Data selection criteria and visualization are described in the Section 2.

An important question for metabolic phenotyping and liver function testing with caffeine is how the coadministration of other drugs and compounds affects caffeine clearance. Consequently, we studied in the second analysis the reported caffeine-drug interactions in the data set. The impact of drugs was quantified using the change in AUC between a coadministration and a respective control $log\left(\frac{AUC_{coadministration}}{AUC_{control}}\right)$ (see Figures 5A,C).

Only case-controlled studies, mostly cross-over trials with a washout phase were included in the analysis. Corresponding controls were not matched across different studies. Overall coadministration data with AUC difference was available for 33 substances in our data set. In accordance with FDA, EMA and PMDA guidelines (Sudsakorn et al., 2020) we classified substances as inhibitors or inducers of caffeine clearance based on changes in AUC: FDA–Clinical DDI guidance: strong, moderate and weak inhibitors increase the AUC ≥5 -fold, ≥2 to <5 -fold, ≥1.25 to <2 -fold, respectively; strong, moderate and weak inducers decrease the AUC by ≥80 percent, ≥50 to <80 percent and ≥20 to <50 percent, respectively.

Most substances do not affect the AUC of caffeine, with the exception of fluvoxamine, pipemidic acid and norfloxacin, which inhibit caffeine clearance. Tipranavir was the only substance showing a weak induction of caffeine clearance, but only in steady state dosing (not after a single dose) (Dumond et al., 2010)). Substances administered as a cocktail along side caffeine, aiming to phenotype several enzymes simultaneously did not affect the AUC of caffeine, substantiating the use of caffeine as part of a drug cocktail design (Turpault et al., 2009; Dumond et al., 2010; Oh et al., 2012; Doroshyenko et al., 2013; Kakuda et al., 2014; Tanaka et al., 2014; Armani et al., 2017; Edwards et al., 2017; Lammers et al., 2018). Our analysis shows that only a minority of studied drugs show an interaction with caffeine, confirming its value for phenotyping even under co-administration. As a side note, the protocols for studying caffeine-drug interactions were highly variable, e.g., the applied caffeine dose and the dose of the coadministrated substance varied between studies. Our results suggest that most medications can be safely consumed in combination with caffeine with exception of the antidepressant fluvoxamine, the antibacterial pipemidic acid and the antibiotic norfloxacin in which case caution is warranted.

### 3.6 Caffeine-Disease Interactions

An important question for using caffeine as a test substance for liver function testing and phenotyping, as well as for drugs metabolized via CYP1A2, is how disease affects the pharmacokinetics and elimination of caffeine. To study this question, we stratified caffeine clearance rates based on the reported disease of subjects and groups in the data set. To quantify the effect of disease the absolute clearance of caffeine (Figure 5B) and the logarithmic difference to a control group $log\left(\frac{AUC_{disease}}{AUC_{control}}\right)$ (Figure 5D) were analyzed. The corresponding controls were not matched across different studies.

None of the reported diseases increased the clearance rate of caffeine. Most of the diseases contained in this data set are diseases of the liver (e.g., alcoholic cirrhosis, primary biliary cholangitis) or are known to affect the liver (e.g., alcohol dependent). Cirrhotic liver disease had moderate to strong effects on the caffeine clearance with large variability in the reported data. Malaria and obesity had no effect on clearance with caffeine. An issue in the study of caffeine-disease interaction is that control group and disease group are different subjects (no cross-over design). In addition diseases were reported very heterogeneously (e.g., either only cirrhosis or with underlying cause such as alcoholic cirrhosis).

### 3.7 Metabolic Phenotyping

**FIGURE 6:**
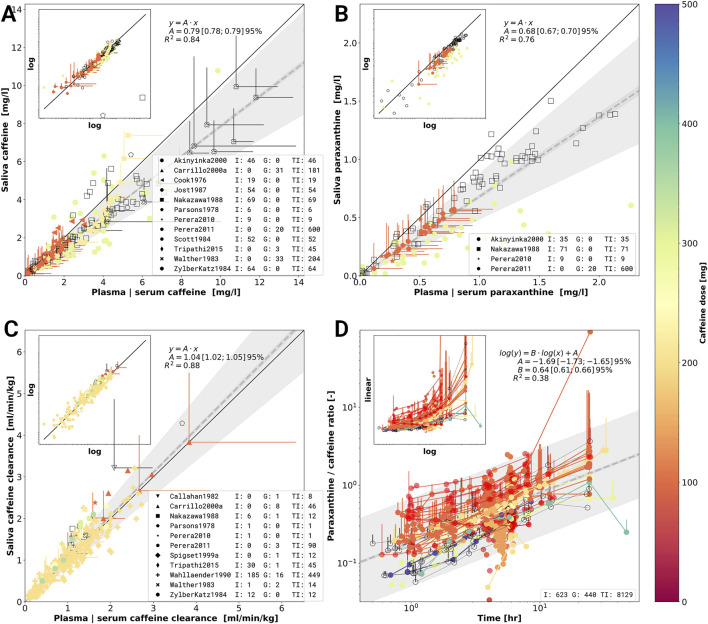
Meta-analysis of caffeine and paraxanthine concentrations in plasma, serum and saliva. **(A)** Caffeine concentrations in saliva versus caffeine concentrations in plasma or serum. Individual data points come from a single investigation taken at identical times after caffeine dosing. Marker shape encodes the different study. Markers are color-coded by caffeine dose in mg. Empty markers correspond to data in which dosage was reported per body weight but no information on the subject weight was available. **(B)** Paraxanthine concentrations in saliva versus paraxanthine concentrations in plasma or serum analogue to **(A)**. **(C)** Caffeine clearance calculated from caffeine concentrations in saliva versus plasma or serum clearance. The panels A, B and C are in linear scale with a log-log inlet showing the same data. The dashed line in A, B and C represents a linear regression (*y* = *A* ⋅ *x*) with wide shaded area being 95% confidence interval of the sample variability and narrow shaded area the 95% confidence interval of the fitted mean of the scaling factor *A*. **(D)** Time dependency of the metabolic ratio paraxanthine/caffeine. Metabolic ratios are measured in plasma, serum, or saliva. Data points belonging to a single time course from a study are connected via a line. The dashed line corresponds to the linear regression log(*y*)= *B* ⋅ log(*t*)+ *A*. Jitter was applied on the time axes for better visibility of overlapping points. Data selection criteria and visualization are described in the Section 2.

An important question for metabolic phenotyping and liver function tests with caffeine is how saliva measurements correlate with plasma or serum caffeine measurements, in the following referred to as blood-based measurements. A good correlation would allow simple non-invasive phenotyping using saliva samples. To study this question we analyzed 1) the relationship of blood-based concentrations of caffeine and paraxanthine with their respective saliva concentrations (Figures 6A,B); and 2) how the caffeine clearance measured in saliva correlate to blood-based measurements (Figure 6C).

Systematic errors due to different dosing protocols and different clinical investigation seem to be minimal as the data from multiple studies shows very consistent results. Linear regressions were performed to quantify the relation between saliva and blood-based caffeine and paraxanthine measurements (see Figures 6A–C). The resulting scaling factors of saliva to blood-based concentration of caffeine and paraxanthine are 0.79 ± 0.01 and 0.68 ± 0.02 ( $\bar{x}\pm \mathrm{SD}$ ), respectively. Pearson correlation coefficients between saliva and blood-based concentrations for caffeine and paraxanthine are 0.84 and 0.76, respectively.

When comparing saliva-based caffeine clearance against blood-based clearance (Figure 6C) an even stronger correlation of 0.88 with a scaling factor of 01.04 ± 0.02 ( $\bar{x}\pm \mathrm{SD}$ ) is observed. The integrated clearance data strongly indicates that clearance can either be calculated from saliva or blood-based measurements.

Paraxanthine/caffeine ratios are mainly used for metabolic phenotyping based on caffeine. Whereas most studies use 6 h to phenotype no clear consensus exists in the literature and metabolic ratios are reported for varying time points after caffeine application. Paraxanthine/caffeine ratios for caffeine administered either as a single dose or in a cocktail to healthy, non-smoking, non-pregnant, and non-oral contraceptive consuming subjects were investigated (Figure 6D). By applying this strict data filtering, the variability due to either smoking and oral contraceptive use (see Section 3.3), caffeine-drug interactions (see Section 3.5) or disease (see Section 3.6) could be removed from the metabolic ratios.

Early and late time-sampling are least suitable for phenotyping. At these time points concentrations are low, resulting in relatively high random errors and thus low single to noise ratio. In an early stage, the outcome of metabolic ratios are further influenced by the distribution phase of the substance and its absorption kinetics, both affected by form and route of administration. Main results are that the metabolic phenotyping with paraxanthine/caffeine ratios is strongly time dependent with increasing ratios with time; and that a clear caffeine-dose dependency exists in the phenotyping with smaller caffeine doses increasing the metabolic ratio. Our results show the importance of clear standardized protocols for metabolic phenotyping.

In summary, by integrating data from multiple studies we could show a very good correlation between saliva and plasma caffeine concentrations, paraxanthine concentrations and pharmacokinetic parameters calculated from saliva versus plasma concentrations. This pooled data provides a strong argument for caffeine phenotyping based on saliva samples. Furthermore, we analyzed the time dependency of paraxanthine/caffeine ratios often used for phenotyping of CYP1A2. Our time dependent correlation allows to correct caffeine/paraxanthine ratios depending on the time after application and caffeine dose.

## 4 Discussion

Within this work, we performed a systematic data integration and multiple data analyses of reported data on caffeine pharmacokinetics in adults focusing on applications in metabolic phenotyping and liver function testing. To our knowledge, this is the largest open pharmacokinetics data set in humans with this kind of data being urgently needed to enable reproducible pharmacokinetics (Ioannidis, 2019). Data integration from multiple sources allows to solidify existing knowledge, increase statistical power, increase generalizability, and create new insights into the relationship between variables (Thacker, 1988). We show for instance, by systematically curating group and subject information on smoking status and oral contraceptive use a reproducible and consistent effect of smoking induction and inhibition via oral contraceptives over many studies and the complete dose regime of applied caffeine. Only a small subset of studies was specifically designed to study these questions (e.g., smoking by (Parsons and Neims, 1978; Benowitz et al., 2003; Backman et al., 2008; Gunes et al., 2009) and oral contraceptives by (Patwardhan et al., 1980; Rietveld et al., 1984; Abernethy and Todd, 1985)). Publicly providing comprehensive pharmacokinetics data in combination with detailed metadata allows to study new aspects of caffeine pharmacokinetics often not even anticipated by the original investigators. One example of such a new aspect is the assessment of dose and time-dependency of metabolic phenotyping via paraxanthine/caffeine ratios even if many of the data sets only report data for a single time point and dose.

Within this work we could confirm that oral contraceptives, smoking and drugs such as fluvoxamine or pipedemic acid alter the pharmacokinetics of caffeine, and that a saliva based metabolic phenotyping approach has very good correlation with blood based approaches, thereby solidifying existing knowledge. In addition novel aspects of the pharmacokinetics of caffeine could be elucidated. By data integration we could not only study the dose dependency of caffeine pharmacokinetics parameters, but also of the effects of smoking and oral contraceptives. For the first time we could show that both the induction of caffeine clearance by smoking and the reduction of caffeine clearance by oral contraceptives is an effect independent of the actual caffeine dose and that caffeine pharmacokinetics is affected by these confounding factors over a wide dose range. Important new results for the metabolic phenotyping with caffeine are our analysis of dose and time-dependency of paraxanthine/caffeine ratios. To our knowledge this time-dose-dependency has not been reported so far, and is especially relevant for phenotyping under very low doses such as cocktail approaches. Based on our results metabolic phenotyping data could be corrected for the dosage and time effects, thereby allowing to integrate data taken under various phenotyping protocols.

The dosage of a substance can have large effects on pharmacokinetic parameters. Using the data set we could evaluate the effect of caffeine dose on pharmacokinetic parameters systematically, e.g., on clearance (Figure 3A, Figure 4A, Figure 5B), half-life (Figure 3B, Figure 4B, Figure 5B), AUC (Figure 5A), or time-dependence of paraxanthine/caffeine ratios used in metabolic phenotyping (Figure 6D) by either directly using dose as a dimension of our analysis or by color coding based on dose. Caffeine dose has strong effects on the AUC of caffeine or the metabolic ratios of paraxanthine/caffeine whereas no clear effects on clearance or half-life could be observed. Our data set and analyses underline the importance of correcting for caffeine dose in the analysis of pharmacokinetic parameters and provide information for dosage corrections.

Various separation methods and detectors (mass spectrometry, UV/Vis, immunoassays or photodiode array) can be used for the quantification of caffeine metabolites and pharmacokinetics. The applied technique and assay can have large confounding effects. For instance cross-reactivity between caffeine and its metabolites have lead to false identification of concentrations by immunoassays (e.g., EMIT, ELISA) (Fligner and Opheim 1988). In earlier studies for example, EMIT methods showed a high cross-reactivity to paraxanthine (28%) Zysset et al. (1984). Newer methods showed low cross reactivity towards paraxanthine (0.08%), however, high cross-reactivity towards theophyline (16%) (Carvalho et al., 2012). Based on our analysis no systematic difference between the reported type of assays could be detected (see Figure 3, Figure 4). This result is limited by the details of reporting with many studies not providing sufficient information on the quantification method.

An important outcome of our analysis are the very good correlations between saliva- and blood-based measurements for caffeine with 0.77 *R* ^2^ = 0.74 in very good agreement with data reported previously in individual studies 0.74 ± 0.1 *R* ^2^ = 0.98 (Akinyinka et al., 2000), 0.79 ± 0.05 *R* ^2^ = 0.96 (Zylber-Katz et al., 1984), 0.74 ± 0.08 (Newton et al., 1981), 0.74 ± 0.08 *R* ^2^ = 0.90 (Jost et al., 1987), 0.71 *R* ^2^ = 0.89 (Scott et al., 1984), 0.73 ± 0.06 (Walther et al., 1983), and for paraxanthine 0.68 *R* ^2^ = 0.76 compared to 0.77 *R* ^2^ = 0.91 (Nakazawa and Tanaka, 1988).

Several studies have shown that blood-derived pharmacokinetic parameters show excellent correlation with saliva-derived parameters (Newton et al., 1981; Akinyinka et al., 2000). We could confirm this observation when systematically analyzing the correlation between saliva- and plasma/serum-derived clearance. By integration of data from multiple studies we could increase the power of the conclusion and show the robustness of the reported correlation.

Overall we could show that the data from multiple studies are in very good agreement with each other after excluding data with confounding factors such as smoking or oral contraceptive use. These integrated results are a strong argument for saliva based metabolic phenotyping and liver function tests with caffeine, with sampling from saliva being convenient, painless, economical, without the requirement for special devices. Further, they allow simple repeated sampling as often required for pharmacokinetic research (Zylber-Katz et al., 1984).

Our systematic curation and analysis of reported caffeine data provided an overview of the current state and limitations of reporting of pharmacokinetic data. In summary, an accepted standard, minimum information guidelines, and standardized meta-data for the reporting of pharmacokinetics data of caffeine are missing. This finding is apparently not only true for the pharmacokinetics of caffeine but rather generally true for reporting of pharmacokinetics research. Major shortcomings in reporting are missing minimum information on factors that are known to influence the pharmacokinetics of caffeine (e.g., smoking and oral contraceptive status). Often not even basic subject information (e.g., weight, sex, or age) are reported. These factors are essential in the analysis of the pharmacokinetics of any substance *in vivo* (Stader et al., 2019). In general-purpose pharmacokinetic data sets, concentration-time profiles are the fundamental and most valuable data type. Common practice however is not to report the raw measured data but only derived pharmacokinetic parameters or metabolic ratios and not on individual participants level. Individual participants data have major advantages (Riley et al., 2010). We strongly advocate the reporting of all data on an individual level while including detailed anonymized meta information alongside the concentration-time profiles. Access to the individual raw data would enable data integration with different data sets and the stratification of the data under various aspects. There are recent efforts in creating a standard resource for that matter (Grzegorzewski et al., 2020).

Data integration and meta-analysis methods may be limited by selection bias, performance bias, detection bias, attrition bias, reporting bias and other biases (Higgins and Collaboration, 2020) but the extent of it in the field of pharmacokinetics is at large unknown (Ioannidis, 2019). Based on our data set we could evaluate the bias due to the type of assay and concluded that no bias could be found. Despite being the most comprehensive analysis so far, we could only present selected aspects mainly driven by the availability of reported data. Our focus in this study was on key factors affecting caffeine pharmacokinetics (smoking and oral contraceptive use), caffeine-drug and caffeine-disease interactions, as well as information relevant for the metabolic phenotyping and liver function testing with caffeine. Other important factors such as the pharmacogenetics of caffeine or urinary metabolic ratios have not been presented. Importantly, the corresponding data was curated and is readily available in PK-DB, but often very sparse (e.g., in case of genetic variants) or very heterogeneous (e.g., in case of urinary data). From our study (Table 1 and other investigations Koonrungsesomboon et al. (2018), it is striking, how little caffeine pharmacogenetics data is captured in the literature despite high heritability of CYP1A2 activity (Rasmussen et al., 2002; Matthaei et al., 2016). Our systematic analysis identified this gap of knowledge and more research in this area is needed.

Despite many implemented measures to ensure high data quality (e.g., validation rules and checking of studies by multiple curators), we are aware that the created data set may contain mistakes. Please report such instances so that these can be resolved.

The scope of the presented data set is limited to caffeine pharmacokinetics in human adults. As identified by the systemic search, the data set is far from comprehensive. At very least further 145 studies are eligible for inclusion, but were classified as low priority for the analyses. Future work will include this data and extend our data curation effort towards children and infants. Beyond the 145 missing studies, additional studies with relevant data exist. Please contact us in such cases so that we can include these additional studies. Contribution of missing data is highly appreciated. Also if you want to contribute a caffeine data set of your own please get in contact.

Importantly, our results are not only applicable to caffeine, but many aspects can be translated to other substances metabolized via CYP1A2, e.g., to the LiMAx liver function tests based on the CYP1A2 substrate methacetin (Rubin et al., 2017). For instance based on our analysis we expect smoking and oral contraceptive use to be confounding factors of LiMAx tests, which should be recorded and be accounted for in the evaluation of liver function.

The large inter-individual variability in caffeine pharmacokinetics is a major limitation for metabolic phenotyping and liver function tests. Our work allowed for the first time to systematically evaluate the effect of key factors on caffeine pharmacokinetics such as smoking, oral contraceptive usage or caffeine-drug interactions. An important next step will be the development of methods to quantify and correct for these confounding factors. This could allow to reduce variability in caffeine based testing. A promising tool in this context are physiological-based pharmacokinetics (PBPK) models (Jones and Rowland-Yeo, 2013) using information from the established data set as input for stratification and individualization. We could recently show that such an approach based on a similar data set for indocyanine green (ICG) allowed to account for important factors affecting ICG based liver function tests (Köller et al., 2021).

## Data Availability

Publicly available datasets were analyzed in this study. This data can be found here: https://pk-db.com.

## References

[B1] Abernethy D. R. Todd E. L. (1985). Impairment of Caffeine Clearance by Chronic Use of Low-Dose Oestrogen-Containing Oral Contraceptives. Eur. J. Clin. Pharmacol. 28, 425–428. 10.1007/BF00544361 4029248

[B2] Abernethy D. R. Todd E. L. Schwartz J. B. (1985). Caffeine Disposition in Obesity. Br. J. Clin. Pharmacol. 20, 61–66. 10.1111/j.1365-2125.1985.tb02799.x 4027137 PMC1400629

[B3] Akinyinka O. O. Sowunmi A. Honeywell R. Renwick A. G. (2000). The Effects of Acute Falciparum Malaria on the Disposition of Caffeine and the Comparison of Saliva and Plasma-Derived Pharmacokinetic Parameters in Adult Nigerians. Eur. J. Clin. Pharmacol. 56, 159–165. 10.1007/s002280050735 10877011

[B4] Amchin J. Zarycranski W. Taylor K. P. Albano D. Klockowski P. M. (1999). Effect of Venlafaxine on Cyp1a2-dependent Pharmacokinetics and Metabolism of Caffeine. J. Clin. Pharmacol. 39, 252–259. 10073324

[B5] Armani S. Ting L. Sauter N. Darstein C. Tripathi A. P. Wang L. (2017). Drug Interaction Potential of Osilodrostat (Lci699) Based on its Effect on the Pharmacokinetics of Probe Drugs of Cytochrome P450 Enzymes in Healthy Adults. Clin. Drug Investig. 37, 465–472. 10.1007/s40261-017-0497-0 PMC5394143 28155129

[B6] Arnaud M. J. (2011). Pharmacokinetics and Metabolism of Natural Methylxanthines in Animal and Man. Handb Exp. Pharmacol. 200, 33–91. 10.1007/978-3-642-13443-2_3 20859793

[B7] Arnaud M. J. Welsch C. (1982). “Theophylline and Caffeine Metabolism in Man,” in Theophylline and other Methylxanthines/Theophyllin und andere Methylxanthine. Methods in Clinical Pharmacology. Editors Rietbrock N. Woodcock B. G. Staib A. H. (Wiesbaden, Germany: Vieweg), 3, 135–148. 10.1007/978-3-663-05268-5_18

[B8] Arold G. Donath F. Maurer A. Diefenbach K. Bauer S. Henneicke-von Zepelin H. H. (2005). No Relevant Interaction with Alprazolam, Caffeine, Tolbutamide, and Digoxin by Treatment with a Low-Hyperforin St John's Wort Extract. Planta Med. 71, 331–337. 10.1055/s-2005-864099 15856409

[B9] Axelrod J. Reichenthal J. (1953). The Fate of Caffeine in Man and a Method for its Estimation in Biological Material. J. Pharmacol. Exp. Ther. 107, 519–523. 13053413

[B10] Azcona O. Barbanoj M. J. Torrent J. Jané F. (1995). Evaluation of the central Effects of Alcohol and Caffeine Interaction. Br. J. Clin. Pharmacol. 40, 393–400. 10.1111/j.1365-2125.1995.tb04562.x 8554942 PMC1365159

[B11] Backman J. T. Schröder M. T. Neuvonen P. J. (2008). Effects of Gender and Moderate Smoking on the Pharmacokinetics and Effects of the Cyp1a2 Substrate Tizanidine. Eur. J. Clin. Pharmacol. 64, 17–24. 10.1007/s00228-007-0389-y 17955229

[B12] Balogh A. Harder S. Vollandt R. Staib A. H. (1992). Intra-individual Variability of Caffeine Elimination in Healthy Subjects. Int. J. Clin. Pharmacol. Ther. Toxicol. 30, 383–387. 1304170

[B13] Balogh A. Klinger G. Henschel L. Börner A. Vollanth R. Kuhnz W. (1995). Influence of Ethinylestradiol-Containing Combination Oral Contraceptives with Gestodene or Levonorgestrel on Caffeine Elimination. Eur. J. Clin. Pharmacol. 48, 161–166. 10.1007/BF00192743 7589032

[B14] Bapiro T. E. Sayi J. Hasler J. A. Jande M. Rimoy G. Masselle A. (2005). Artemisinin and Thiabendazole Are Potent Inhibitors of Cytochrome P450 1a2 (Cyp1a2) Activity in Humans. Eur. J. Clin. Pharmacol. 61, 755–761. 10.1007/s00228-005-0037-3 16261361

[B15] Barnett G. Segura J. de la Torre R. Carbó M. (1990). Pharmacokinetic Determination of Relative Potency of Quinolone Inhibition of Caffeine Disposition. Eur. J. Clin. Pharmacol. 39, 63–69. 10.1007/BF02657060 2177401

[B16] Bchir F. Dogui M. Ben Fradj R. Arnaud M. J. Saguem S. (2006). Differences in Pharmacokinetic and Electroencephalographic Responses to Caffeine in Sleep-Sensitive and Non-sensitive Subjects. C R. Biol. 329, 512–519. 10.1016/j.crvi.2006.01.006 16797457

[B17] Beach C. A. Mays D. C. Guiler R. C. Jacober C. H. Gerber N. (1986). Inhibition of Elimination of Caffeine by Disulfiram in normal Subjects and Recovering Alcoholics. Clin. Pharmacol. Ther. 39, 265–270. 10.1038/clpt.1986.37 3948467

[B18] Becker A. B. Simons K. J. Gillespie C. A. Simons F. E. (1984). The Bronchodilator Effects and Pharmacokinetics of Caffeine in Asthma. N. Engl. J. Med. 310, 743–746. 10.1056/NEJM198403223101202 6700656

[B19] Begas E. Kouvaras E. Tsakalof A. Papakosta S. Asprodini E. K. (2007). *In Vivo* evaluation of Cyp1a2, Cyp2a6, Nat-2 and Xanthine Oxidase Activities in a Greek Population Sample by the Rp-Hplc Monitoring of Caffeine Metabolic Ratios. Biomed. Chromatogr. 21, 190–200. 10.1002/bmc.736 17221922

[B20] Begas E. Kouvaras E. Tsakalof A. K. Bounitsi M. Asprodini E. K. (2015). Development and Validation of a Reversed-phase Hplc Method for Cyp1a2 Phenotyping by Use of a Caffeine Metabolite Ratio in Saliva. Biomed. Chromatogr. 29, 1657–1663. 10.1002/bmc.3475 25891161

[B21] Benowitz N. L. Jacob P. Mayan H. Denaro C. (1995). Sympathomimetic Effects of Paraxanthine and Caffeine in Humans. Clin. Pharmacol. Ther. 58, 684–691. 10.1016/0009-9236(95)90025-X 8529334

[B22] Benowitz N. L. Peng M. Jacob P. (2003). Effects of Cigarette Smoking and Carbon Monoxide on Chlorzoxazone and Caffeine Metabolism. Clin. Pharmacol. Ther. 74, 468–474. 10.1016/j.clpt.2003.07.001 14586387

[B23] Birkett D. J. Miners J. O. (1991). Caffeine Renal Clearance and Urine Caffeine Concentrations during Steady State Dosing. Implications for Monitoring Caffeine Intake during Sports Events. Br. J. Clin. Pharmacol. 31, 405–408. 10.1111/j.1365-2125.1991.tb05553.x 2049248 PMC1368325

[B24] Blanchard J. Sawers S. J. (1983b). Comparative Pharmacokinetics of Caffeine in Young and Elderly Men. J. Pharmacokinet. Biopharm. 11, 109–126. 10.1007/BF01061844 6886969

[B25] Blanchard J. Sawers S. J. Jonkman J. H. Tang-Liu D. D. (1985). Comparison of the Urinary Metabolite Profile of Caffeine in Young and Elderly Males. Br. J. Clin. Pharmacol. 19, 225–232. 10.1111/j.1365-2125.1985.tb02635.x 3986081 PMC1463701

[B26] Blanchard J. Sawers S. J. (1983c). Relationship between Urine Flow Rate and Renal Clearance of Caffeine in Man. J. Clin. Pharmacol. 23, 134–138. 10.1002/j.1552-4604.1983.tb02716.x 6863577

[B27] Blanchard J. Sawers S. J. (1983a). The Absolute Bioavailability of Caffeine in Man. Eur. J. Clin. Pharmacol. 24, 93–98. 10.1007/BF00613933 6832208

[B28] Bonati M. Latini R. Galletti F. Young J. F. Tognoni G. Garattini S. (1982). Caffeine Disposition after Oral Doses. Clin. Pharmacol. Ther. 32, 98–106. 10.1038/clpt.1982.132 7083737

[B29] Bozikas V. P. Papakosta M. Niopas I. Karavatos A. Mirtsou-Fidani V. (2004). Smoking Impact on Cyp1a2 Activity in a Group of Patients with Schizophrenia. Eur. Neuropsychopharmacol. 14, 39–44. 10.1016/s0924-977x(03)00061-0 14659985

[B30] Brazier J. L. Descotes J. Lery N. Ollagnier M. Evreux J. C. (1980). Inhibition by Idrocilamide of the Disposition of Caffeine. Eur. J. Clin. Pharmacol. 17, 37–43. 10.1007/BF00561675 7371698

[B31] Broughton L. J. Rogers H. J. (1981). Decreased Systemic Clearance of Caffeine Due to Cimetidine. Br. J. Clin. Pharmacol. 12, 155–159. 10.1111/j.1365-2125.1981.tb01194.x 7306430 PMC1401851

[B32] Bruce M. Scott N. Lader M. Marks V. (1986). The Psychopharmacological and Electrophysiological Effects of Single Doses of Caffeine in Healthy Human Subjects. Br. J. Clin. Pharmacol. 22, 81–87. 10.1111/j.1365-2125.1986.tb02883.x 3741730 PMC1401080

[B33] Calatayud O. Rodríguez M. Sánchez-Alcaraz A. Ibáñez P. (1995). Caffeine Test Assessment for Measuring Liver Function in Critically Ill Patients. J. Clin. Pharm. Ther. 20, 23–29. 10.1111/j.1365-2710.1995.tb00621.x 7775610

[B34] Callahan M. M. Robertson R. S. Arnaud M. J. Branfman A. R. McComish M. F. Yesair D. W. (1982). Human Metabolism of [1-methyl-14c]- and [2-14c]caffeine after Oral Administration. Drug Metab. Dispos 10, 417–423. 6126344

[B35] Callahan M. M. Robertson R. S. Branfman A. R. McComish M. F. Yesair D. W. (1983). Comparison of Caffeine Metabolism in Three Nonsmoking Populations after Oral Administration of Radiolabeled Caffeine. Drug Metab. Dispos 11, 211–217. 6135578

[B36] Campbell M. E. Spielberg S. P. Kalow W. (1987). A Urinary Metabolite Ratio that Reflects Systemic Caffeine Clearance. Clin. Pharmacol. Ther. 42, 157–165. 10.1038/clpt.1987.126 3608349

[B37] Carbó M. Segura J. De la Torre R. Badenas J. M. Camí J. (1989). Effect of Quinolones on Caffeine Disposition. Clin. Pharmacol. Ther. 45, 234–240. 10.1038/clpt.1989.23 2920498

[B38] Carrillo J. A. Christensen M. Ramos S. I. Alm C. Dahl M. L. Benitez J. (2000). Evaluation of Caffeine as an *In Vivo* Probe for Cyp1a2 Using Measurements in Plasma, Saliva, and Urine. Ther. Drug Monit. 22, 409–417. 10.1097/00007691-200008000-00008 10942180

[B39] Carvalho J. J. Weller M. G. Panne U. Schneider R. J. (2012). Monitoring Caffeine in Human Saliva Using a Newly Developed ELISA. Anal. Lett. 45, 2549–2561. 10.1080/00032719.2012.696226

[B40] Chen Y. Liu W. H. Chen B. L. Fan L. Han Y. Wang G. (2010). Plant Polyphenol Curcumin Significantly Affects Cyp1a2 and Cyp2a6 Activity in Healthy, Male Chinese Volunteers. Ann. Pharmacother. 44, 1038–1045. 10.1345/aph.1M533 20484172

[B41] Chen Y. Tu J. H. He Y. J. Zhang W. Wang G. Tan Z. R. (2009a). Effect of Sodium Tanshinone Ii a Sulfonate on the Activity of Cyp1a2 in Healthy Volunteers. Xenobiotica 39, 508–513. 10.1080/00498250902951763 19534587

[B42] Chen Y. Xiao P. Ou-Yang D. S. Fan L. Guo D. Wang Y. N. (2009b). Simultaneous Action of the Flavonoid Quercetin on Cytochrome P450 (Cyp) 1a2, Cyp2a6, N-Acetyltransferase and Xanthine Oxidase Activity in Healthy Volunteers. Clin. Exp. Pharmacol. Physiol. 36, 828–833. 10.1111/j.1440-1681.2009.05158.x 19215233

[B43] Cheng W. S. Murphy T. L. Smith M. T. Cooksley W. G. Halliday J. W. Powell L. W. (1990). Dose-dependent Pharmacokinetics of Caffeine in Humans: Relevance as a Test of Quantitative Liver Function. Clin. Pharmacol. Ther. 47, 516–524. 10.1038/clpt.1990.66 2328560

[B44] Chia H. Y. Yau W. P. Ho H. K. (2016). Establishing Population Distribution of Drug-Metabolizing Enzyme Activities for the Use of Salivary Caffeine as a Dynamic Liver Function Marker in a Singaporean Chinese Population. Biopharm. Drug Dispos 37, 168–181. 10.1002/bdd.2006 26862045

[B45] Christensen M. Tybring G. Mihara K. Yasui-Furokori N. Carrillo J. A. Ramos S. I. (2002). Low Daily 10-mg and 20-mg Doses of Fluvoxamine Inhibit the Metabolism of Both Caffeine (Cytochrome P4501a2) and Omeprazole (Cytochrome P4502c19). Clin. Pharmacol. Ther. 71, 141–152. 10.1067/mcp.2002.121788 11907488

[B46] Cook C. E. Tallent C. R. Amerson E. W. Myers M. W. Kepler J. A. Taylor G. F. (1976). Caffeine in Plasma and Saliva by a Radioimmunoassay Procedure. J. Pharmacol. Exp. Ther. 199, 679–686. 1033273

[B47] Cornish H. H. Christman A. A. (1957). A Study of the Metabolism of Theobromine, Theophylline, and Caffeine in Man. J. Biol. Chem. 228, 315–323. 10.1016/s0021-9258(18)70714-x 13475320

[B48] Culm-Merdek K. E. von Moltke L. L. Harmatz J. S. Greenblatt D. J. (2005). Fluvoxamine Impairs Single-Dose Caffeine Clearance without Altering Caffeine Pharmacodynamics. Br. J. Clin. Pharmacol. 60, 486–493. 10.1111/j.1365-2125.2005.02467.x 16236038 PMC1884944

[B49] Cysneiros R. M. Farkas D. Harmatz J. S. von Moltke L. L. Greenblatt D. J. (2007). Pharmacokinetic and Pharmacodynamic Interactions between Zolpidem and Caffeine. Clin. Pharmacol. Ther. 82, 54–62. 10.1038/sj.clpt.6100211 17443132

[B50] Darwish M. Kirby M. Robertson P. Hellriegel E. T. (2008). Interaction Profile of Armodafinil with Medications Metabolized by Cytochrome P450 Enzymes 1a2, 3a4 and 2c19 in Healthy Subjects. Clin. Pharmacokinet. 47, 61–74. 10.2165/00003088-200847010-00006 18076219

[B51] Desmond P. V. Patwardhan R. V. Johnson R. F. Schenker S. (1980). Impaired Elimination of Caffeine in Cirrhosis. Dig. Dis. Sci. 25, 193–197. 10.1007/BF01308138 7371463

[B52] Dinger J. Woods C. Brandt S. D. Meyer M. R. Maurer H. H. (2016). Cytochrome P450 Inhibition Potential of New Psychoactive Substances of the Tryptamine Class. Toxicol. Lett. 241, 82–94. 10.1016/j.toxlet.2015.11.013 26599973

[B53] Djordjevic N. Ghotbi R. Bertilsson L. Jankovic S. Aklillu E. (2008). Induction of Cyp1a2 by Heavy Coffee Consumption in Serbs and Swedes. Eur. J. Clin. Pharmacol. 64, 381–385. 10.1007/s00228-007-0438-6 18157525

[B54] Doroshyenko O. Rokitta D. Zadoyan G. Klement S. Schläfke S. Dienel A. (2013). Drug Cocktail Interaction Study on the Effect of the Orally Administered Lavender Oil Preparation Silexan on Cytochrome P450 Enzymes in Healthy Volunteers. Drug Metab. Dispos 41, 987–993. 10.1124/dmd.112.050203 23401474

[B55] Drozdzik M. Busch D. Lapczuk J. Müller J. Ostrowski M. Kurzawski M. (2018). Protein Abundance of Clinically Relevant Drug-Metabolizing Enzymes in the Human Liver and Intestine: A Comparative Analysis in Paired Tissue Specimens. Clin. Pharmacol. Ther. 104, 515–524. 10.1002/cpt.967 29205295

[B56] Dumond J. B. Vourvahis M. Rezk N. L. Patterson K. B. Tien H. C. White N. (2010). A Phenotype-Genotype Approach to Predicting Cyp450 and P-Glycoprotein Drug Interactions with the Mixed Inhibitor/inducer Tipranavir/ritonavir. Clin. Pharmacol. Ther. 87, 735–742. 10.1038/clpt.2009.253 20147896 PMC2882206

[B57] Edwards J. E. Eliot L. Parkinson A. Karan S. MacConell L. (2017). Assessment of Pharmacokinetic Interactions between Obeticholic Acid and Caffeine, Midazolam, Warfarin, Dextromethorphan, Omeprazole, Rosuvastatin, and Digoxin in Phase 1 Studies in Healthy Subjects. Adv. Ther. 34, 2120–2138. 10.1007/s12325-017-0601-0 28808886 PMC5599467

[B58] Faber M. S. Fuhr U. (2004). Time Response of Cytochrome P450 1a2 Activity on Cessation of Heavy Smoking. Clin. Pharmacol. Ther. 76, 178–184. 10.1016/j.clpt.2004.04.003 15289794

[B59] Faber M. S. Jetter A. Fuhr U. (2005). Assessment of Cyp1a2 Activity in Clinical Practice: Why, How, and when? Basic Clin. Pharmacol. Toxicol. 97, 125–134. 10.1111/j.1742-7843.2005.pto_973160.x 16128905

[B60] Fligner C. L. Opheim K. E. (1988). Caffeine and its Dimethylxanthine Metabolites in Two Cases of Caffeine Overdose: a Cause of Falsely Elevated Theophylline Concentrations in Serum. J. Anal. Toxicol. 12, 339–343. 10.1093/jat/12.6.339 3072449

[B61] Frary C. D. Johnson R. K. Wang M. Q. (2005). Food Sources and Intakes of Caffeine in the Diets of Persons in the united states. J. Am. Diet. Assoc. 105, 110–113. 10.1016/j.jada.2004.10.027 15635355

[B62] Fuhr U. Klittich K. Staib A. H. (1993). Inhibitory Effect of Grapefruit Juice and its Bitter Principal, Naringenin, on Cyp1a2 Dependent Metabolism of Caffeine in Man. Br. J. Clin. Pharmacol. 35, 431–436. 10.1111/j.1365-2125.1993.tb04162.x 8485024 PMC1381556

[B63] Fuhr U. Rost K. L. Engelhardt R. Sachs M. Liermann D. Belloc C. (1996). Evaluation of caffeine as a test drug for cyp1a2, nat2 and cyp2e1 phenotyping in man by *In Vivo* versus *In Vitro* correlations. Pharmacogenetics 6, 159–176. 10.1097/00008571-199604000-00003 9156694

[B64] Furge L. L. Fletke K. J. (2007). Hplc Determination of Caffeine and Paraxanthine in Urine: An Assay for Cytochrome P450 1a2 Activity. Biochem. Mol. Biol. Educ. 35, 138–144. 10.1002/bmb.28 21591074

[B65] George J. Murphy T. Roberts R. Cooksley W. G. Halliday J. W. Powell L. W. (1986). Influence of Alcohol and Caffeine Consumption on Caffeine Elimination. Clin. Exp. Pharmacol. Physiol. 13, 731–736. 10.1111/j.1440-1681.1986.tb02414.x 3802578

[B66] Ghassabian S. Chetty M. Tattam B. N. Chem M. C. Glen J. Rahme J. (2009). A High-Throughput Assay Using Liquid Chromatography-Tandem Mass Spectrometry for Simultaneous *In Vivo* Phenotyping of 5 Major Cytochrome P450 Enzymes in Patients. Ther. Drug Monit. 31, 239–246. 10.1097/FTD.0b013e318197e1bf 19307938

[B67] Ghotbi R. Christensen M. Roh H. K. Ingelman-Sundberg M. Aklillu E. Bertilsson L. (2007). Comparisons of Cyp1a2 Genetic Polymorphisms, Enzyme Activity and the Genotype-Phenotype Relationship in Swedes and Koreans. Eur. J. Clin. Pharmacol. 63, 537–546. 10.1007/s00228-007-0288-2 17370067

[B68] Gilbert R. (1984). The Methylxanthine Beverages and Foods: Chemistry, Consumption, and Health Effects. Prog. Clin. Biol. Res. 158, 1–413. 6527996

[B69] Gonzalez Hernandez F. Carter S. J. Iso-Sipilä J. Goldsmith P. Almousa A. A. Gastine S. (2021). An Automated Approach to Identify Scientific Publications Reporting Pharmacokinetic Parameters. Wellcome Open Res. 6, 88. 10.12688/wellcomeopenres.16718.1 34381873 PMC8343403

[B70] Granfors M. T. Backman J. T. Laitila J. Neuvonen P. J. (2005). Oral Contraceptives Containing Ethinyl Estradiol and Gestodene Markedly Increase Plasma Concentrations and Effects of Tizanidine by Inhibiting Cytochrome P450 1a2. Clin. Pharmacol. Ther. 78, 400–411. 10.1016/j.clpt.2005.06.009 16198659

[B71] Grzegorzewski J. Brandhorst J. Green K. Eleftheriadou D. Duport Y. Barthorscht F. (2020). Pk-db: Pharmacokinetics Database for Individualized and Stratified Computational Modeling. Nucleic Acids Res. 49, D1358–D1364. 10.1093/nar/gkaa990 PMC7779054 33151297

[B72] Gunes A. Dahl M. L. (2008). Variation in Cyp1a2 Activity and its Clinical Implications: Influence of Environmental Factors and Genetic Polymorphisms. Pharmacogenomics 9, 625–637. 10.2217/14622416.9.5.625 18466106

[B73] Gunes A. Ozbey G. Vural E. H. Uluoglu C. Scordo M. G. Zengil H. (2009). Influence of Genetic Polymorphisms, Smoking, Gender and Age on Cyp1a2 Activity in a Turkish Population. Pharmacogenomics 10, 769–778. 10.2217/pgs.09.22 19450128

[B74] Gurley B. J. Gardner S. F. Hubbard M. A. Williams D. K. Gentry W. B. Cui Y. (2005a). Clinical Assessment of Effects of Botanical Supplementation on Cytochrome P450 Phenotypes in the Elderly: St John's Wort, Garlic Oil, Panax Ginseng and Ginkgo Biloba. Drugs Aging 22, 525–539. 10.2165/00002512-200522060-00006 15974642 PMC1858666

[B75] Gurley B. J. Gardner S. F. Hubbard M. A. Williams D. K. Gentry W. B. Khan I. A. (2005b). *In Vivo* effects of goldenseal, kava kava, black cohosh, and valerian on human cytochrome p450 1a2, 2d6, 2e1, and 3a4/5 phenotypes. Clin. Pharmacol. Ther. 77, 415–426. 10.1016/j.clpt.2005.01.009 15900287 PMC1894911

[B76] Hakooz N. M. (2009). Caffeine Metabolic Ratios for the *In Vivo* Evaluation of Cyp1a2, N-Acetyltransferase 2, Xanthine Oxidase and Cyp2a6 Enzymatic Activities. Curr. Drug Metab. 10, 329–338. 10.2174/138920009788499003 19519341

[B77] Haller C. A. Jacob P. Benowitz N. L. (2002). Pharmacology of Ephedra Alkaloids and Caffeine after Single-Dose Dietary Supplement Use. Clin. Pharmacol. Ther. 71, 421–432. 10.1067/mcp.2002.124523 12087345

[B78] Hamon-Vilcot B. Simon T. Becquemont L. Poirier J. M. Piette F. Jaillon P. (2004). Effects of Malnutrition on Cytochrome P450 1a2 Activity in Elderly Patients. Therapie 59, 247–251. 10.2515/therapie:2004048 15359622

[B79] Harder S. Fuhr U. Staib A. H. Wolff T. (1989). Ciprofloxacin-caffeine: a Drug Interaction Established Using *In Vivo* and *In Vitro* Investigations. Am. J. Med. 87, 89S–91S. 10.1016/0002-9343(89)90031-4 2589393

[B80] Harder S. Staib A. H. Beer C. Papenburg A. Stille W. Shah P. M. (1988). 4-quinolones Inhibit Biotransformation of Caffeine. Eur. J. Clin. Pharmacol. 35, 651–656. 10.1007/BF00637602 2853056

[B81] Hashiguchi M. Fujimura A. Ohashi K. Ebihara A. (1992). Diurnal Effect on Caffeine Clearance. J. Clin. Pharmacol. 32, 184–187. 10.1002/j.1552-4604.1992.tb03824.x 1613129

[B82] He H. Ma D. Crone L. B. Butawan M. Meibohm B. Bloomer R. J. (2017). Assessment of the Drug-Drug Interaction Potential between Theacrine and Caffeine in Humans. J. Caffeine Res. 7, 95–102. 10.1089/jcr.2017.0006 28875060 PMC5582588

[B83] Healy D. P. Polk R. E. Kanawati L. Rock D. T. Mooney M. L. (1989). Interaction between Oral Ciprofloxacin and Caffeine in normal Volunteers. Antimicrob. Agents Chemother. 33, 474–478. 10.1128/aac.33.4.474 2729942 PMC172463

[B84] Healy D. P. Schoenle J. R. Stotka J. Polk R. E. (1991). Lack of Interaction between Lomefloxacin and Caffeine in normal Volunteers. Antimicrob. Agents Chemother. 35, 660–664. 10.1128/aac.35.4.660 2069371 PMC245075

[B85] Hetzler R. K. Knowlton R. G. Somani S. M. Brown D. D. Perkins R. M. (19901985). Effect of Paraxanthine on Ffa Mobilization after Intravenous Caffeine Administration in Humans. J. Appl. Physiol. (1985) 68, 44–47. 10.1152/jappl.1990.68.1.44 2312486

[B86] Holstege A. Kurz M. Weinbeck M. Gerok W. (1993). Excretion of Caffeine and its Primary Degradation Products into Bile. J. Hepatol. 17, 67–73. 10.1016/s0168-8278(05)80523-9 8445222

[B87] Holstege A. Staiger M. Haag K. Gerok W. (1989). Correlation of Caffeine Elimination and Child's Classification in Liver Cirrhosis. Klin Wochenschr 67, 6–15. 10.1007/BF01736528 2921843

[B88] Ioannidis J. P. A. (2019). Reproducible Pharmacokinetics. J. Pharmacokinet. Pharmacodyn 46, 111–116. 10.1007/s10928-019-09621-y 31004315

[B89] Jeppesen U. Loft S. Poulsen H. E. Brśen K. (1996). A Fluvoxamine-Caffeine Interaction Study. Pharmacogenetics 6, 213–222. 10.1097/00008571-199606000-00003 8807660

[B90] Jiang Z. Dragin N. Jorge-Nebert L. F. Martin M. V. Guengerich F. P. Aklillu E. (2006). Search for an Association between the Human Cyp1a2 Genotype and Cyp1a2 Metabolic Phenotype. Pharmacogenet Genomics 16, 359–367. 10.1097/01.fpc.0000204994.99429.46 16609368

[B91] Jodynis-Liebert J. Flieger J. Matuszewska A. Juszczyk J. (2004). Serum Metabolite/caffeine Ratios as a Test for Liver Function. J. Clin. Pharmacol. 44, 338–347. 10.1177/0091270004263468 15051740

[B92] Joeres R. Klinker H. Heusler H. Epping J. Richter E. (1987). Influence of Mexiletine on Caffeine Elimination. Pharmacol. Ther. 33, 163–169. 10.1016/0163-7258(87)90046-5 3628469

[B93] Joeres R. Klinker H. Heusler H. Epping J. Zilly W. Richter E. (1988). Influence of Smoking on Caffeine Elimination in Healthy Volunteers and in Patients with Alcoholic Liver Cirrhosis. Hepatology 8, 575–579. 10.1002/hep.1840080323 3371873

[B94] Jones H. Rowland-Yeo K. (2013). Basic Concepts in Physiologically Based Pharmacokinetic Modeling in Drug Discovery and Development. CPT Pharmacometrics Syst. Pharmacol. 2, e63. 10.1038/psp.2013.41 23945604 PMC3828005

[B95] Jost G. Wahlländer A. von Mandach U. Preisig R. (1987). Overnight Salivary Caffeine Clearance: a Liver Function Test Suitable for Routine Use. Hepatology 7, 338–344. 10.1002/hep.1840070221 3557314

[B96] Higgins J. P. T. Collaboration C. (Editors) (2020). Cochrane Handbook for Systematic Reviews of Interventions. second edition edn (Hoboken, NJ: Wiley-Blackwell).

[B97] Kakuda T. N. Van Solingen-Ristea R. M. Onkelinx J. Stevens T. Aharchi F. De Smedt G. (2014). The Effect of Single- and Multiple-Dose Etravirine on a Drug Cocktail of Representative Cytochrome P450 Probes and Digoxin in Healthy Subjects. J. Clin. Pharmacol. 54, 422–431. 10.1002/jcph.214 24165884

[B98] Kalow W. Tang B. K. (1993). The Use of Caffeine for Enzyme Assays: a Critical Appraisal. Clin. Pharmacol. Ther. 53, 503–514. 10.1038/clpt.1993.63 8491061

[B99] Kamimori G. H. Joubert A. Otterstetter R. Santaromana M. Eddington N. D. (1999). The Effect of the Menstrual Cycle on the Pharmacokinetics of Caffeine in normal, Healthy Eumenorrheic Females. Eur. J. Clin. Pharmacol. 55, 445–449. 10.1007/s002280050654 10492057

[B100] Kamimori G. H. Karyekar C. S. Otterstetter R. Cox D. S. Balkin T. J. Belenky G. L. (2002). The Rate of Absorption and Relative Bioavailability of Caffeine Administered in Chewing Gum versus Capsules to normal Healthy Volunteers. Int. J. Pharm. 234, 159–167. 10.1016/s0378-5173(01)00958-9 11839447

[B101] Kamimori G. H. Somani S. M. Knowlton R. G. Perkins R. M. (1987). The Effects of Obesity and Exercise on the Pharmacokinetics of Caffeine in Lean and Obese Volunteers. Eur. J. Clin. Pharmacol. 31, 595–600. 10.1007/BF00606637 3830245

[B102] Kaplan G. B. Greenblatt D. J. Ehrenberg B. L. Goddard J. E. Cotreau M. M. Harmatz J. S. (1997). Dose-dependent Pharmacokinetics and Psychomotor Effects of Caffeine in Humans. J. Clin. Pharmacol. 37, 693–703. 10.1002/j.1552-4604.1997.tb04356.x 9378841

[B103] Kinzig-Schippers M. Fuhr U. Zaigler M. Dammeyer J. Rüsing G. Labedzki A. (1999). Interaction of Pefloxacin and Enoxacin with the Human Cytochrome P450 Enzyme Cyp1a2. Clin. Pharmacol. Ther. 65, 262–274. 10.1016/S0009-9236(99)70105-0 10096258

[B104] Klein K. Winter S. Turpeinen M. Schwab M. Zanger U. M. (2010). Pathway-targeted Pharmacogenomics of Cyp1a2 in Human Liver. Front. Pharmacol. 1, 129. 10.3389/fphar.2010.00129 21918647 PMC3171976

[B105] Koch J. P. ten Tusscher G. W. Koppe J. G. Guchelaar H. J. (1999). Validation of a High-Performance Liquid Chromatography Assay for Quantification of Caffeine and Paraxanthine in Human Serum in the Context of Cyp1a2 Phenotyping. Biomed. Chromatogr. 13, 309–314. 10.1002/(SICI)1099-0801(199906)13:4<309:AID-BMC881>3.0.CO;2-J 10416066

[B106] Köller A. Grzegorzewski J. Tautenhahn H.-M. König M. (2021). Prediction of Survival after Partial Hepatectomy Using a Physiologically Based Pharmacokinetic Model of Indocyanine green Liver Function Tests. bioRxiv. 10.1101/2021.06.15.448411 PMC8646028 34880771

[B107] Koonrungsesomboon N. Khatsri R. Wongchompoo P. Teekachunhatean S. (2018). The Impact of Genetic Polymorphisms on CYP1A2 Activity in Humans: A Systematic Review and Meta-Analysis. Pharmacogenomics J. 18, 760–768. 10.1038/s41397-017-0011-3 29282363

[B108] Kot M. Daniel W. A. (2008). Caffeine as a Marker Substrate for Testing Cytochrome P450 Activity in Human and Rat. Pharmacol. Rep. 60, 789–797. 19211970

[B109] Kukongviriyapan V. Senggunprai L. Prawan A. Gaysornsiri D. Kukongviriyapan U. Aiemsa-Ard J. (2004). Salivary Caffeine Metabolic Ratio in Alcohol-dependent Subjects. Eur. J. Clin. Pharmacol. 60, 103–107. 10.1007/s00228-004-0734-3 15022032

[B110] Laizure S. C. Meibohm B. Nelson K. Chen F. Hu Z. Y. Parker R. B. (2017). Comparison of Caffeine Disposition Following Administration by Oral Solution (Energy Drink) and Inspired Powder (Aeroshot) in Human Subjects. Br. J. Clin. Pharmacol. 83, 2687–2694. 10.1111/bcp.13389 28758694 PMC5698589

[B111] Lammers L. A. Achterbergh R. Romijn J. A. Mathôt R. A. A. (2018). Short-term Fasting Alters Pharmacokinetics of Cytochrome P450 Probe Drugs: Does Protein Binding Play a Role? Eur. J. Drug Metab. Pharmacokinet. 43, 251–257. 10.1007/s13318-017-0437-7 28929443 PMC5854751

[B112] Lane J. D. Steege J. F. Rupp S. L. Kuhn C. M. (1992). Menstrual Cycle Effects on Caffeine Elimination in the Human Female. Eur. J. Clin. Pharmacol. 43, 543–546. 10.1007/BF02285099 1483492

[B113] Lane S. D. Green C. E. Schmitz J. M. Rathnayaka N. Fang W. B. Ferré S. (2014). Comparison of Caffeine and D-Amphetamine in Cocaine-dependent Subjects: Differential Outcomes on Subjective and Cardiovascular Effects, Reward Learning, and Salivary Paraxanthine. J. Addict. Res. Ther. 5, 176. 10.4172/2155-6105.1000176 25414797 PMC4235768

[B114] Lelo A. Birkett D. J. Robson R. A. Miners J. O. (1986a). Comparative Pharmacokinetics of Caffeine and its Primary Demethylated Metabolites Paraxanthine, Theobromine and Theophylline in Man. Br. J. Clin. Pharmacol. 22, 177–182. 10.1111/j.1365-2125.1986.tb05246.x 3756065 PMC1401099

[B115] Lelo A. Miners J. O. Robson R. A. Birkett D. J. (1986b). Quantitative Assessment of Caffeine Partial Clearances in Man. Br. J. Clin. Pharmacol. 22, 183–186. 10.1111/j.1365-2125.1986.tb05247.x 3756066 PMC1401107

[B116] Levy M. Zylber-Katz E. (1983). Caffeine Metabolism and Coffee-Attributed Sleep Disturbances. Clin. Pharmacol. Ther. 33, 770–775. 10.1038/clpt.1983.105 6851408

[B117] Liberati A. Altman D. G. Tetzlaff J. Mulrow C. Gøtzsche P. C. Ioannidis J. P. (2009). The PRISMA Statement for Reporting Systematic Reviews and Meta-Analyses of Studies that Evaluate Healthcare Interventions: Explanation and Elaboration. BMJ 339, b2700. 10.1136/bmj.b2700 19622552 PMC2714672

[B118] Magnusson M. O. Dahl M. L. Cederberg J. Karlsson M. O. Sandström R. (2008). Pharmacodynamics of Carbamazepine-Mediated Induction of Cyp3a4, Cyp1a2, and Pgp as Assessed by Probe Substrates Midazolam, Caffeine, and Digoxin. Clin. Pharmacol. Ther. 84, 52–62. 10.1038/sj.clpt.6100431 17971810

[B119] Marks V. Kelly J. F. (1973). Absorption of Caffeine from tea, Coffee, and Coca Cola. Lancet 1, 827. 10.1016/s0140-6736(73)90625-9 4121243

[B120] Matthaei J. Tzvetkov M. V. Strube J. Sehrt D. Sachse-Seeboth C. Hjelmborg J. B. (2016). Heritability of Caffeine Metabolism: Environmental Effects Masking Genetic Effects on Cyp1a2 Activity but Not on Nat2. Clin. Pharmacol. Ther. 100, 606–616. 10.1002/cpt.444 27509179

[B121] May D. C. Jarboe C. H. VanBakel A. B. Williams W. M. (1982). Effects of Cimetidine on Caffeine Disposition in Smokers and Nonsmokers. Clin. Pharmacol. Ther. 31, 656–661. 10.1038/clpt.1982.91 7075114

[B122] McDonagh J. E. Nathan V. V. Bonavia I. C. Moyle G. R. Tanner A. R. (1991). Caffeine Clearance by Enzyme Multiplied Immunoassay Technique: a Simple, Inexpensive, and Useful Indicator of Liver Function. Gut 32, 681–684. 10.1136/gut.32.6.681 2060878 PMC1378889

[B123] McLean C. Graham T. E. (2002). Effects of Exercise and thermal Stress on Caffeine Pharmacokinetics in Men and Eumenorrheic Women. J. Appl. Physiol. 93, 1471–1478. 10.1152/japplphysiol.00762.2000 12235049

[B124] McQuilkin S. H. Nierenberg D. W. Bresnick E. (1995). Analysis of Within-Subject Variation of Caffeine Metabolism when Used to Determine Cytochrome P4501a2 and N-Acetyltransferase-2 Activities. Cancer Epidemiol. Biomarkers Prev. 4, 139–146. 7742721

[B125] Millard J. T. Passang T. Ye J. Kline G. M. Beachy T. M. Hepburn V. L. (2018). Genotype and Phenotype of Caffeine Metabolism: A Biochemistry Laboratory experiment. J. Chem. Educ. 95, 1856–1860. 10.1021/acs.jchemed.8b00318

[B126] Miners J. O. Birkett D. J. (1996). The Use of Caffeine as a Metabolic Probe for Human Drug Metabolizing Enzymes. Gen. Pharmacol. 27, 245–249. 10.1016/0306-3623(95)02014-4 8919637

[B127] Mitchell D. C. Knight C. A. Hockenberry J. Teplansky R. Hartman T. J. (2014). Beverage Caffeine Intakes in the u.S. Food Chem. Toxicol. 63, 136–142. 10.1016/j.fct.2013.10.042 24189158

[B128] Moonen H. J. Moonen E. J. Maas L. Dallinga J. W. Kleinjans J. C. de Kok T. M. (2004). CYP1A2 and NAT2 Genotype/phenotype Relations and Urinary Excretion of 2-Amino-1-Methyl-6-Phenylimidazo[4,5-B]pyridine (PhIP) in a Human Dietary Intervention Study. Food Chem. Toxicol. 42, 869–878. 10.1016/j.fct.2004.01.010 15110095

[B129] Murphy T. L. McIvor C. Yap A. Cooksley W. G. Halliday J. W. Powell L. W. (1988). The Effect of Smoking on Caffeine Elimination: Implications for its Use as a Semiquantitative Test of Liver Function. Clin. Exp. Pharmacol. Physiol. 15, 9–13. 10.1111/j.1440-1681.1988.tb01003.x 2482799

[B130] Muscat J. E. Pittman B. Kleinman W. Lazarus P. Stellman S. D. Richie J. P. (2008). Comparison of Cyp1a2 and Nat2 Phenotypes between Black and white Smokers. Biochem. Pharmacol. 76, 929–937. 10.1016/j.bcp.2008.07.024 18703023 PMC2597011

[B131] Myrand S. P. Sekiguchi K. Man M. Z. Lin X. Tzeng R. Y. Teng C. H. (2008). Pharmacokinetics/genotype Associations for Major Cytochrome P450 Enzymes in Native and First- and Third-Generation Japanese Populations: Comparison with Korean, Chinese, and Caucasian Populations. Clin. Pharmacol. Ther. 84, 347–361. 10.1038/sj.clpt.6100482 18231117

[B132] Nakazawa K. Tanaka H. (1988). pharmacokinetics of Caffeine and Dimethylxanthines in Plasma and Saliva. Yakugaku Zasshi 108, 653–658. 10.1248/yakushi1947.108.7_653 3241301

[B133] Nehlig A. (2018). Interindividual Differences in Caffeine Metabolism and Factors Driving Caffeine Consumption. Pharmacol. Rev. 70, 384–411. 10.1124/pr.117.014407 29514871

[B134] Newton R. Broughton L. J. Lind M. J. Morrison P. J. Rogers H. J. Bradbrook I. D. (1981). Plasma and Salivary Pharmacokinetics of Caffeine in Man. Eur. J. Clin. Pharmacol. 21, 45–52. 10.1007/BF00609587 7333346

[B135] Oh K. S. Park S. J. Shinde D. D. Shin J. G. Kim D. H. (2012). High-sensitivity Liquid Chromatography-Tandem Mass Spectrometry for the Simultaneous Determination of Five Drugs and Their Cytochrome P450-specific Probe Metabolites in Human Plasma. J. Chromatogr. B Analyt Technol. Biomed. Life Sci. 895-896, 56–64. 10.1016/j.jchromb.2012.03.014 22483397

[B136] Park G. J. Katelaris P. H. Jones D. B. Seow F. Le Couteur D. G. Ngu M. C. (2003). Validity of the 13c-Caffeine Breath Test as a Noninvasive, Quantitative Test of Liver Function. Hepatology 38, 1227–1236. 10.1053/jhep.2003.50475 14578861

[B137] Parsons W. D. Neims A. H. (1978). Effect of Smoking on Caffeine Clearance. Clin. Pharmacol. Ther. 24, 40–45. 10.1002/cpt197824140 657717

[B138] Patwardhan R. V. Desmond P. V. Johnson R. F. Schenker S. (1980). Impaired Elimination of Caffeine by Oral Contraceptive Steroids. J. Lab. Clin. Med. 95, 603–608. 7359014

[B139] Perera V. Gross A. S. McLachlan A. J. (2010). Caffeine and Paraxanthine Hplc Assay for Cyp1a2 Phenotype Assessment Using Saliva and Plasma. Biomed. Chromatogr. 24, 1136–1144. 10.1002/bmc.1419 20853468

[B140] Perera V. Gross A. S. Xu H. McLachlan A. J. (2011). Pharmacokinetics of Caffeine in Plasma and Saliva, and the Influence of Caffeine Abstinence on Cyp1a2 Metrics. J. Pharm. Pharmacol. 63, 1161–1168. 10.1111/j.2042-7158.2011.01326.x 21827488

[B141] Priller S. (2005). Cytochrome P450 1a2 Phenotyping for Student Laboratories. Pharm. Education J. 10.1080/15602210500193219

[B142] Puri B. K. Heard C. R. Monro J. A. (2020). Is There a Sex Difference in Adult Salivary Clearance of Caffeine (1,3,7-Trimethylpurine-2,6-Dione)? J. Oral Biol. Craniofac. Res. 10, 20–22. 10.1016/j.jobcr.2020.01.010 32071850 PMC7016269

[B143] Rasmussen B. B. Brix T. H. Kyvik K. O. Brøsen K. (2002). The Interindividual Differences in the 3-demthylation of Caffeine Alias Cyp1a2 Is Determined by Both Genetic and Environmental Factors. Pharmacogenetics 12, 473–478. 10.1097/00008571-200208000-00008 12172216

[B144] Renner E. Wietholtz H. Huguenin P. Arnaud M. J. Preisig R. (1984). Caffeine: a Model Compound for Measuring Liver Function. Hepatology 4, 38–46. 10.1002/hep.1840040107 6420303

[B145] Rietveld E. C. Broekman M. M. Houben J. J. Eskes T. K. van Rossum J. M. (1984). Rapid Onset of an Increase in Caffeine Residence Time in Young Women Due to Oral Contraceptive Steroids. Eur. J. Clin. Pharmacol. 26, 371–373. 10.1007/BF00548769 6734698

[B146] Riley R. D. Lambert P. C. Abo-Zaid G. (2010). Meta-analysis of Individual Participant Data: Rationale, Conduct, and Reporting. BMJ 340, c221. 10.1136/bmj.c221 20139215

[B147] Rubin T. M. Heyne K. Luchterhand A. Jan Bednarsch J. W R Vondran F. Polychronidis G. (2017). Kinetic Validation of the LiMAx Test during 10 000 Intravenous 13C-Methacetin Breath Tests. J. Breath Res. 12, 016005. 10.1088/1752-7163/aa820b 28742055

[B148] Sadek P. Pan X. Shepherd P. Malandain E. Carney J. Coleman H. (2017). A Randomized, Two-Way Crossover Study to Evaluate the Pharmacokinetics of Caffeine Delivered Using Caffeinated Chewing Gum versus a Marketed Caffeinated Beverage in Healthy Adult Volunteers. J. Caffeine Res. 7, 125–132. 10.1089/jcr.2017.0025 29230348 PMC5724581

[B149] Schmider J. Brockmöller J. Arold G. Bauer S. Roots I. (1999). Simultaneous Assessment of Cyp3a4 and Cyp1a2 Activity *In Vivo* with Alprazolam and Caffeine. Pharmacogenetics 9, 725–734. 10.1097/01213011-199912000-00007 10634135

[B150] Schrenk D. Brockmeier D. Mörike K. Bock K. W. Eichelbaum M. (1998). A Distribution Study of Cyp1a2 Phenotypes Among Smokers and Non-smokers in a Cohort of Healthy Caucasian Volunteers. Eur. J. Clin. Pharmacol. 53, 361–367. 10.1007/s002280050394 9516038

[B151] Scott N. R. Chakraborty J. Marks V. (1984). Determination of Caffeine, Theophylline and Theobromine in Serum and Saliva Using High-Performance Liquid Chromatography. Ann. Clin. Biochem. 21 (Pt 2), 120–124. 10.1177/000456328402100208 6712142

[B152] Scott N. R. Stambuk D. Chakraborty J. Marks V. Morgan M. Y. (1989). The Pharmacokinetics of Caffeine and its Dimethylxanthine Metabolites in Patients with Chronic Liver Disease. Br. J. Clin. Pharmacol. 27, 205–213. 10.1111/j.1365-2125.1989.tb05352.x 2713214 PMC1379781

[B153] Scott N. R. Stambuk D. Chakraborty J. Marks V. Morgan M. Y. (1988). Caffeine Clearance and Biotransformation in Patients with Chronic Liver Disease. Clin. Sci. (Lond) 74, 377–384. 10.1042/cs0740377 3356110

[B154] Seng K. Y. Fun C. Y. Law Y. L. Lim W. M. Fan W. Lim C. L. (2009). Population Pharmacokinetics of Caffeine in Healthy Male Adults Using Mixed-Effects Models. J. Clin. Pharm. Ther. 34, 103–114. 10.1111/j.1365-2710.2008.00976.x 19125908

[B155] Soto J. Alsar M. J. Sacristan J. A. (1995). Assessment of the Time Course of Drugs with Inhibitory Effects on Hepatic Metabolic Activity Using Successive Salivary Caffeine Tests. Pharmacotherapy 15, 781–784. 8602388

[B156] Spigset O. Hägg S. Söderström E. Dahlqvist R. (1999). The Paraxanthine:caffeine Ratio in Serum or in Saliva as a Measure of Cyp1a2 Activity: when Should the Sample Be Obtained? Pharmacogenetics 9, 409–412. 10.1097/00008571-199906000-00019 10471076

[B157] Stader F. Siccardi M. Battegay M. Kinvig H. Penny M. A. Marzolini C. (2019). Repository Describing an Aging Population to Inform Physiologically Based Pharmacokinetic Models Considering Anatomical, Physiological, and Biological Age-dependent Changes. Clin. Pharmacokinet. 58, 483–501. 10.1007/s40262-018-0709-7 30128967

[B158] Stille W. Harder S. Mieke S. Beer C. Shah P. M. Frech K. (1987). Decrease of Caffeine Elimination in Man during Co-administration of 4-quinolones. J. Antimicrob. Chemother. 20, 729–734. 10.1093/jac/20.5.729 3480885

[B159] Sudsakorn S. Bahadduri P. Fretland J. Lu C. (2020). 2020 Fda Drug-Drug Interaction Guidance: A Comparison Analysis and Action Plan by Pharmaceutical Industrial Scientists. Curr. Drug Metab. 21, 403–426. 10.2174/1389200221666200620210522 32562522

[B160] Syed S. A. Kamimori G. H. Kelly W. Eddington N. D. (2005). Multiple Dose Pharmacokinetics of Caffeine Administered in Chewing Gum to normal Healthy Volunteers. Biopharm. Drug Dispos 26, 403–409. 10.1002/bdd.469 16158445

[B161] Tanaka E. Ishikawa A. Yamamoto Y. Osada A. Tsuji K. Fukao K. (1993). Comparison of Hepatic Drug-Oxidizing Activity after Simultaneous Administration of Two Probe Drugs, Caffeine and Trimethadione, to Human Subjects. Pharmacol. Toxicol. 72, 31–33. 10.1111/j.1600-0773.1993.tb01335.x 8441739

[B162] Tanaka S. Uchida S. Inui N. Takeuchi K. Watanabe H. Namiki N. (2014). Simultaneous Lc-Ms/ms Analysis of the Plasma Concentrations of a Cocktail of 5 Cytochrome P450 Substrate Drugs and Their Metabolites. Biol. Pharm. Bull. 37, 18–25. 10.1248/bpb.b13-00401 24389476

[B163] Tang-Liu D. D. Williams R. L. Riegelman S. (1983). Disposition of Caffeine and its Metabolites in Man. J. Pharmacol. Exp. Ther. 224, 180–185. 6848742

[B164] Tantcheva-Poór I. Zaigler M. Rietbrock S. Fuhr U. (1999). Estimation of Cytochrome P-450 Cyp1a2 Activity in 863 Healthy Caucasians Using a Saliva-Based Caffeine Test. Pharmacogenetics 9, 131–144. 10376760

[B165] Thacker S. B. (1988). Meta-analysis. A Quantitative Approach to Research Integration. JAMA 259, 1685–1689. 10.1001/jama.259.11.1685 3278147

[B166] Tian D. D. Natesan S. White J. R. Paine M. F. Paine M. F. (2019). Effects of Common Cyp1a2 Genotypes and Other Key Factors on Intraindividual Variation in the Caffeine Metabolic Ratio: An Exploratory Analysis. Clin. Transl Sci. 12, 39–46. 10.1111/cts.12598 30387917 PMC6342244

[B167] Trang J. M. Blanchard J. Conrad K. A. Harrison G. G. (1985). Relationship between Total Body Clearance of Caffeine and Urine Flow Rate in Elderly Men. Biopharm. Drug Dispos 6, 51–56. 10.1002/bdd.2510060107 3986300

[B168] Trang J. M. Blanchard J. Conrad K. A. Harrison G. G. (1982). The Effect of Vitamin C on the Pharmacokinetics of Caffeine in Elderly Men. Am. J. Clin. Nutr. 35, 487–494. 10.1093/ajcn/35.3.487 7064899

[B169] Tricco A. C. Lillie E. Zarin W. O'Brien K. K. Colquhoun H. Levac D. (2018). PRISMA Extension for Scoping Reviews (PRISMA-ScR): Checklist and Explanation. Ann. Intern. Med. 169, 467–473. 10.7326/M18-0850 30178033

[B170] Tripathi A. Tiwari B. Patil R. Khanna V. Singh V. (2015). The Role of Salivary Caffeine Clearance in the Diagnosis of Chronic Liver Disease. J. Oral Biol. Craniofac. Res. 5, 28–33. 10.1016/j.jobcr.2014.12.003 25853045 PMC4382505

[B171] Turpault S. Brian W. Van Horn R. Santoni A. Poitiers F. Donazzolo Y. (2009). Pharmacokinetic Assessment of a Five-Probe Cocktail for Cyps 1a2, 2c9, 2c19, 2d6 and 3a. Br. J. Clin. Pharmacol. 68, 928–935. 10.1111/j.1365-2125.2009.03548.x 20002088 PMC2810805

[B172] Urry E. Jetter A. Landolt H. P. (2016). Assessment of Cyp1a2 Enzyme Activity in Relation to Type-2 Diabetes and Habitual Caffeine Intake. Nutr. Metab. (Lond) 13, 66. 10.1186/s12986-016-0126-6 27713762 PMC5052791

[B173] Wahlländer A. Mohr S. Paumgartner G. (1990). Assessment of Hepatic Function. Comparison of Caffeine Clearance in Serum and Saliva during the Day and at Night. J. Hepatol. 10, 129–137. 10.1016/0168-8278(90)90041-o 2185297

[B174] Walther H. Banditt P. Köhler E. (1983). Significance of Caffeine Values in Serum, Saliva and Urine-Ddetermination of Pharmacokinetic Data by Non-invasive Methods in Psychopharmacologic Studies. Pharmacopsychiatria 16, 166–170. 10.1055/s-2007-1019492 6657740

[B175] Wang T. Kleber G. Stellaard F. Paumgartner G. (1985). Caffeine Elimination: a Test of Liver Function. Klin Wochenschr 63, 1124–1128. 10.1007/BF02291094 4079279

[B176] White J. R. Padowski J. M. Zhong Y. Chen G. Luo S. Lazarus P. (2016). Pharmacokinetic Analysis and Comparison of Caffeine Administered Rapidly or Slowly in Coffee Chilled or Hot versus Chilled Energy Drink in Healthy Young Adults. Clin. Toxicol. 54, 308–312. 10.3109/15563650.2016.1146740 PMC4898153 27100333

[B177] Yamazaki T. Desai A. Goldwater R. Han D. Howieson C. Akhtar S. (2017). Pharmacokinetic Effects of Isavuconazole Coadministration with the Cytochrome P450 Enzyme Substrates Bupropion, Repaglinide, Caffeine, Dextromethorphan, and Methadone in Healthy Subjects. Clin. Pharmacol. Drug Dev. 6, 54–65. 10.1002/cpdd.281 27273149 PMC5297975

[B178] Yang X. Zhang B. Molony C. Chudin E. Hao K. Zhu J. (2010). Systematic Genetic and Genomic Analysis of Cytochrome P450 Enzyme Activities in Human Liver. Genome Res. 20, 1020–1036. 10.1101/gr.103341.109 20538623 PMC2909567

[B179] Yesair D. W. Branfman A. R. Callahan M. M. (1984). Human Disposition and Some Biochemical Aspects of Methylxanthines. Prog. Clin. Biol. Res. 158, 215–233. 6396646

[B180] Yubero-Lahoz S. Pardo R. Farre M. Mathuna B. Ó. Torrens M. Mustata C. (2012). Changes in Cyp1a2 Activity in Humans after 3,4-methylenedioxymethamphetamine (Mdma, Ecstasy) Administration Using Caffeine as a Probe Drug. Drug Metab. Pharmacokinet. 27, 605–613. 10.2133/dmpk.dmpk-12-rg-032 22673010

[B181] Zanger U. M. Schwab M. (2013). Cytochrome P450 Enzymes in Drug Metabolism: Regulation of Gene Expression, Enzyme Activities, and Impact of Genetic Variation. Pharmacol. Ther. 138, 103–141. 10.1016/j.pharmthera.2012.12.007 23333322

[B182] Zylber-Katz E. Granit L. Levy M. (1984). Relationship between Caffeine Concentrations in Plasma and Saliva. Clin. Pharmacol. Ther. 36, 133–137. 10.1038/clpt.1984.151 6734043

[B183] Zysset T. Wahlländer A. Preisig R. (1984). Evaluation of Caffeine Plasma Levels by an Automated Enzyme Immunoassay (EMIT) in Comparison with a High-Performance Liquid Chromatographic Method. Ther. Drug Monit. 6, 348–354. 10.1097/00007691-198409000-00016 6390797

